# Comparative 4D Label-Free Quantitative Proteomic Analysis of *Bombus terrestris* Provides Insights into Proteins and Processes Associated with Diapause

**DOI:** 10.3390/ijms25010326

**Published:** 2023-12-26

**Authors:** Yan Liu, Long Su, Ruijuan Wang, Xiaoyan Dai, Xiuxue Li, Yuqing Chang, Shan Zhao, Hao Chen, Zhenjuan Yin, Guang’an Wu, Hao Zhou, Li Zheng, Yifan Zhai

**Affiliations:** 1 Institute of Plant Protection, Shandong Academy of Agricultural Sciences, 23788 Gongye North Road, Jinan 250100, China; liuyan8882@126.com (Y.L.); rizhaosulong@163.com (L.S.); wangruijuan1020@126.com (R.W.); 15169087554@163.com (X.D.); xw1904298926@163.com (X.L.); cyq27271@163.com (Y.C.); 15062276975@163.com (S.Z.); cha.active@163.com (H.C.); yinzhenjuan1220@126.com (Z.Y.); zhengli64@126.com (L.Z.); 2 Key Laboratory of Natural Enemies Insects, Ministry of Agriculture and Rural Affairs, Jinan 250100, China; wguangan@163.com (G.W.); zhouenhao@126.com (H.Z.); 3 Shandong Provincial Engineering Technology Research Center on Biocontrol of Crops Pests, Jinan 250100, China

**Keywords:** *Bombus terrestris*, 4D label-free quantitative proteomics, protein abundance, pre-diapause, diapause, post-diapause

## Abstract

Diapause, an adaptative strategy for survival under harsh conditions, is a dynamic multi-stage process. *Bombus terrestris*, an important agricultural pollinator, is declining in the wild, but artificial breeding is possible by imitating natural conditions. Mated queen bees enter reproductive diapause in winter and recover in spring, but the regulatory mechanisms remain unclear. Herein, we conducted a comparative 4D label-free proteomic analysis of queen bees during artificial breeding at seven timepoints, including pre-diapause, diapause, and post-diapause stages. Through bioinformatics analysis of proteomic and detection of substance content changes, our results found that, during pre-diapause stages, queen bees had active mitochondria with high levels of oxidative phosphorylation, high body weight, and glycogen and TAG content, all of which support energy consumption during subsequent diapause. During diapause stages, body weight and water content were decreased but glycerol increased, contributing to cold resistance. Dopamine content, immune defense, and protein phosphorylation were elevated, while fat metabolism, protein export, cell communication, signal transduction, and hydrolase activity decreased. Following diapause termination, JH titer, water, fatty acid, and pyruvate levels increased, catabolism, synaptic transmission, and insulin signaling were stimulated, ribosome and cell cycle proteins were upregulated, and cell proliferation was accelerated. Meanwhile, TAG and glycogen content decreased, and ovaries gradually developed. These findings illuminate changes occurring in queen bees at different diapause stages during commercial production.

## 1. Introduction

Diapause enables insects to survive in unfavorable seasons and synchronizes the reproductive stage and generation of insects with favorable seasonal conditions, thus effectively utilizing resources. Diapause is of great significance in the life cycle of insects and involves ‘toolkit’ genes that modulate development, starvation resistance, stress tolerance, longevity, and reproduction [1,2,3,4]. Diapause is a dynamic process consisting of several successive phases, generally divided into three stages: pre-diapause, diapause, and post-diapause [5]. Pre-diapause can be divided into induction and preparation phases. In pre-diapause, insects perceive specific environmental diapause induction signals, such as temperature and photoperiod, and decide whether to enter diapause after signal transformation (hormone synthesis and secretion), then initiate appropriate preparations for entry into diapause. Diapause stages have three eco-physiological sub-phases: initiation, maintenance, and termination. At this stage, development is gradually arrested and maintained in a specific state until environmental conditions become suitable, at which point diapause is terminated. At the post-diapause stage, if the environmental conditions are suitable, metabolism gradually increases and normal development is resumed. However, if the environmental conditions do not meet their developmental needs, the insects remain in a lower metabolic state and development ceases until the environmental conditions become suitable [5].

Diapause also comes at a cost. On the one hand, the timing of diapause is very strict; if affected by environmental factors, insects enter diapause prematurely, or diapause cannot be terminated in time, this results in a long diapause duration during which the insects may miss the chance to mate or lay eggs, and the metabolic cost of maintaining diapause and the potential risks associated with diapause survival are also increased; if diapause lasts too long or not long enough, insects may be exposed to environmental conditions that are unfavorable to survival [6]. On the other hand, insects generally do not feed or move during diapause; hence, following exposure to low temperatures during overwintering, inactive diapause individuals are easy prey, and large quantities of metabolic reserves of overwintering individuals may be consumed, decreasing the survival rate of insects [7]. Therefore, in addition to diapause itself, both successful entry into diapause and termination of diapause are important for insects.

The buff-tailed bumblebee, *Bombus terrestris* (L.) (Hymenoptera: Apidae), an annual primitively eusocial insect, is an evolutionary intermediate between solitary and higher social bees. Its colonies grow from spring through late summer, and each has a single queen and many workers. As the colony ages, new queens and males are produced, and mated new queens enter reproductive diapause to survive winter, then establish new colonies the following spring [8]. *B. terrestris* are important pollinators of a variety of plants, especially legumes and Solanaceae, because their tongues are longer than those of honeybees, making them better at pollinating flowers with deep corollas [9]. *B. terrestris* has been mass-reared year-round and used commercially in greenhouses for crop pollination since the 1980s, and this species is used worldwide to increase the yield and quality of crops. A thorough understanding of the diapause mechanism is therefore of great significance for large-scale year-round breeding of bumblebees [10,11]. Changes in the *B. terrestris* transcriptome indicate that diapause is associated with nutrient storage, stress resistance, core metabolism, hormone signaling, and various cellular pathways [2].

While transcriptomics provides valuable insight into regulatory mechanisms, mRNA expression levels are often poorly to moderately correlated with protein abundance. Proteins are the biochemically functional components that dictate the adaptability and survival of an organism, particularly under stressful conditions [12]. In recent years, proteomics technology has gradually improved and is now widely used in various fields, including medicine, zoology, botany, and microbiology. Proteomics technology has been applied to the study of various insects. Two-dimensional electrophoresis was used to identify diapause-associated proteins and eight proteins were characterized in the female ladybird, *Coccinella septempunctata* L. [13]. ITRAQ proteomic analysis was employed to analyze the differentially abundant proteins (DAPs) during the pre-diapause, diapause, and post-diapause stages during summer diapause of adult *Galeruca daurica* [14]. Tandem mass tags labeling proteomic analysis was conducted to analyze the metabolic regulation of adult reproductive diapause in *Drosophila suzukii* females and *B. terrestris* [10,11,15]. Label-free quantitative proteomics revealed molecular evidence for the consequences and benefits of mating, as well as novel insights into preparation, survival, and recovering from diapause in *B. terrestris* queen hemolymph, but only 129 proteins were identified [16]. Previous studies on diapausing bumblebees are limited to a few stages or a single tissue. The development of four-dimensional label-free quantification (4D-LFQ) proteomics using a timsTOF Pro mass spectrometry (MS) system is a momentous breakthrough in proteomics technology; it is faster, more sensitive, and achieves superior analytical performance compared with other approaches [17]. Ion mobility was incorporated into MS as a fourth separation dimension in addition to time, *m*/*z*, and ion strength [17,18]. However, this technology has not been used to investigate diapause.

Herein, 4D-LFQ with LC–MS/MS was used to explore the changes in protein abundance during seven stages of diapause in *B. terrestris*. We artificially divided these seven stages into three periods: (a) pre-diapause, mated (M), and before diapause (BD); (b) diapause, diapause for 1 week (D1W), diapause for 12 weeks (D12W), and before diapause termination (BDT); and (c) post-diapause, post-diapause 48 h (PD48h), and post-diapause oviposition (OPD). Few studies have examined the dynamic processes of protein abundance, particularly those related to nutrient accumulation, metabolic activity, and hormone changes in pre-diapause, diapause, and post-diapause queens. This study aimed to provide insight into how diapausing queens prepare for, survive, and recover from diapause.

## 2. Results

### 2.1. Related Protein Profiles in Seven Stages Regarding 4D-LFQ Proteomic Analyses of B. terrestris Queen Diapause

**Figure 1:**
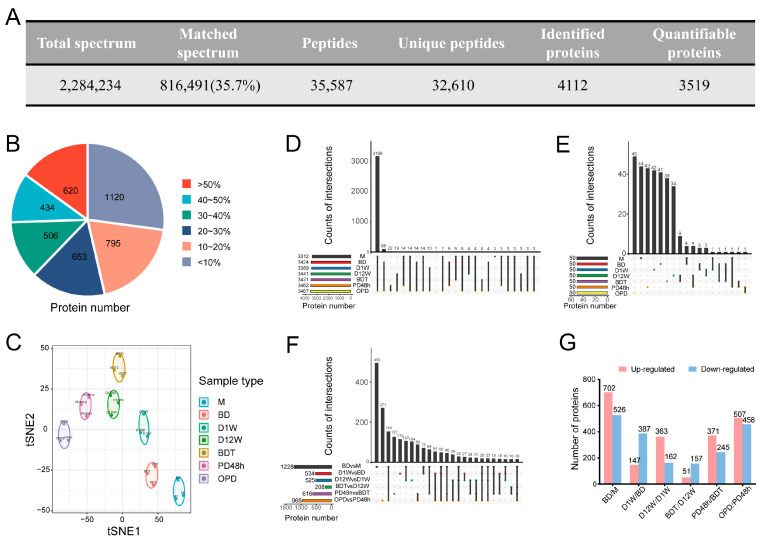
Overall proteomic analyses of *Bombus terrestris* queens during adult reproductive diapause. (**A**) Basic proteome information. (**B**) Distribution of protein sequence coverage; the numbers in the pie chart represent the number of proteins with the same color of sequence coverage. (**C**) tSNE plot of samples (*n* = 3 for each treatment). The upset plot shows the numbers and intersections of identified proteins (**D**), the top 50 most abundant proteins (**E**), and DAPs for six group comparisons (**F**) from different *B. terrestris* proteomes. (**G**) Histogram of upregulated (red) and downregulated (blue) DAPs proteins for six group comparisons.

The 4D-LFQ method was used to investigate and compare differences in protein abundance of the seven diapause stages of *B. terrestris*. In total, 2,284,234 secondary spectra were obtained from mass spectrometry analysis, and, after searching the protein theoretical data library, the effective number of spectra was acquired as 816,491, and the utilization rate of the spectra was 35.7% (Figure 1A). Spectral analysis identified 35,587 peptides, of which 32,610 were unique, and 4112 proteins were identified by proteomics, of which 3519 were quantifiable (Figure 1A, Supplementary Table S1). In total, 73% of the proteins had sequence coverage > 10%, and 19% of the proteins had sequence coverage between 10% and 20% (Figure 1B). The molecular weights of the identified proteins mostly ranged from 10 to 100 kDa, and most corresponded to more than two peptides, indicating the accuracy and credibility of the quantitative results (Supplementary Figure S1A,B). T-distributed Stochastic Neighbor Embedding (t-SNE) was used to assess protein intra-group repeatability. The results of biological replicates were statistically consistent, and all seven groups were clearly distinguished from each other (Figure 1C). We compared overlapping proteins in each group using upset plots, which yielded 3198 common proteins in seven groups, accounting for 78% (3198/4112) of all the identified proteins (Figure 1D). The distribution of the top 50 most abundant proteins in each group was analyzed, and most were unique to groups (Figure 1E). Gene Ontology (GO) analyses of the top 50 proteins in each group were preformed to better understand the physiological processes likely to occur during the different diapause stages. The results showed that the most enriched GO terms in biological process, cellular component, and molecular function in M were immune-related: BD was a mitochondrial-related process, D1W was mitochondrial-related and involved cell morphogenesis, D12W involved cell–cell junction, BDT was a neurotransmitter biosynthetic process and regulation of cellular catabolic process, PD48h was a regulation of cell cycle process, and OPD involved lipid metabolism (Supplementary Figure S2 and Table S2).

DAPs between samples were considered significantly up- and downregulated when the LFQ intensity ratio fold was ≥1.5 or ≤0.67 (*p* < 0.05). As can be seen, BDT vs. D12W had the fewest DAPs, while BD vs. M had the most. Finally, BD vs. M and OPD vs. PD48h had the highest number of common DAPs at 154 (Figure 1F,G).

### 2.2. DAP Analysis

**Figure 2:**
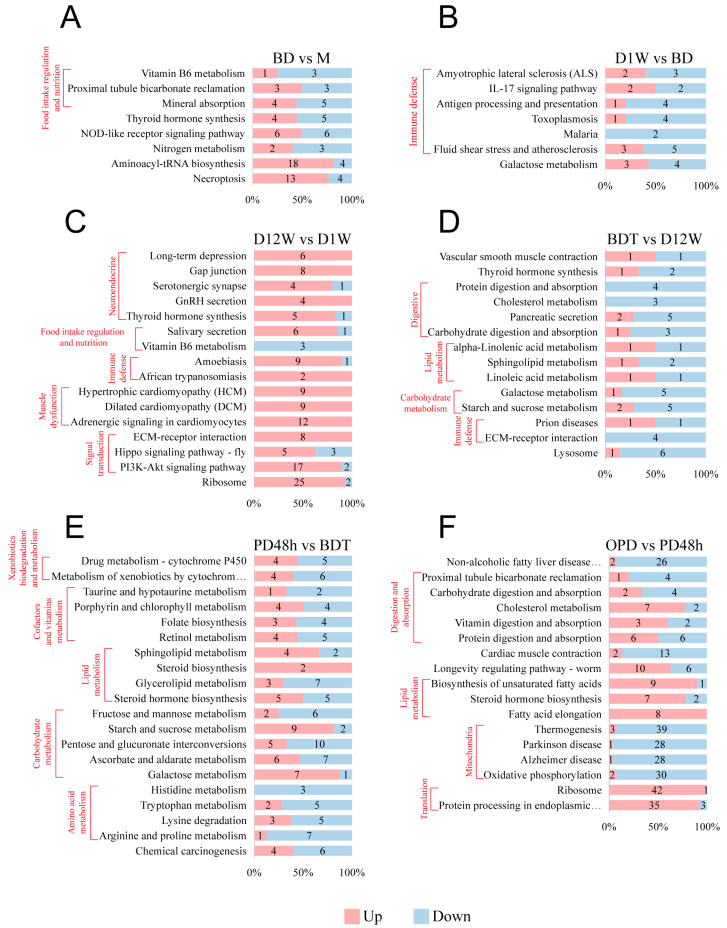
KEGG pathway enrichment analyses of DAPs of *Bombus terrestris* in each comparison group. (**A**) BD vs. M. (**B**) D1W vs. BD. (**C**) D12W vs. D1W. (**D**) BDT vs. D12W. (**E**) PD 48 h vs. BDT. (**F**) OPD vs. PD 48 h. Red represents upregulated proteins, blue represents downregulated proteins, and numbers in diagrams represent DAP numbers.

Kyoto Encyclopedia of Genes and Genomes (KEGG) enrichment analysis was conducted for DAPs in each group comparison. In the BD vs. M comparison, 1228 DAPs (702 up- and 526 downregulated, Figure 1G) were enriched in eight KEGG pathways (*p* < 0.05; Figure 2A). Three pathways were related to digestion and excretion. In the D1W vs. BD comparison, 534 DAPs (147 up- and 387 downregulated, Figure 1G) were enriched in seven KEGG pathways (*p* < 0.05; Figure 2B). Six of these pathways were related to immune defense. In the D12W vs. D1W comparison, 525 DAPs (363 up- and 162 downregulated, Figure 1G) were enriched in 16 KEGG pathways (*p* < 0.05; Figure 2C). Among the sixteen pathways, the representative subclasses included neuroendocrine (five pathways), digestion (two pathways), immune defense (two pathways), muscle dysfunction (three pathways), signal transduction (three pathways), and protein translation (one pathway). In the BDT vs. D12W comparison, 208 DAPs (51 up- and 157 downregulated, Figure 1G) were enriched in 14 KEGG pathways (*p* < 0.05; Figure 2D). Representative subclasses included digestive (five pathways), lipid metabolism (three pathways), carbohydrate metabolism (two pathways), and immune defense (two pathways). In the PD48h vs. BDT comparison, 616 DAPs (371 up- and 245 downregulated, Figure 1G) were enriched in 20 KEGG pathways (*p* < 0.05; Figure 2E). Among the 20 pathways, 19 were related to metabolism, including lipid metabolism, carbohydrate metabolism, amino acid metabolism, xenobiotics and drug metabolism, and cofactor metabolism. In the OPD vs. PD48h comparison, 965 DAPs (507 up- and 458 downregulated, Figure 1G) were enriched in 17 KEGG pathways (*p* < 0.05; Figure 2F). Representative subclasses included digestive, lipid metabolism, and translation, in which the number of upregulated proteins was greater than the number of downregulated proteins. By contrast, the number of downregulated proteins exceeded the number of upregulated proteins in the processes of thermogenesis, Parkinson’s disease, Alzheimer’s disease, and oxidative phosphorylation, indicating dysfunctional mitochondria.

### 2.3. Temporal Expression Patterns of Proteins in Different Diapause Stages

**Figure 3:**
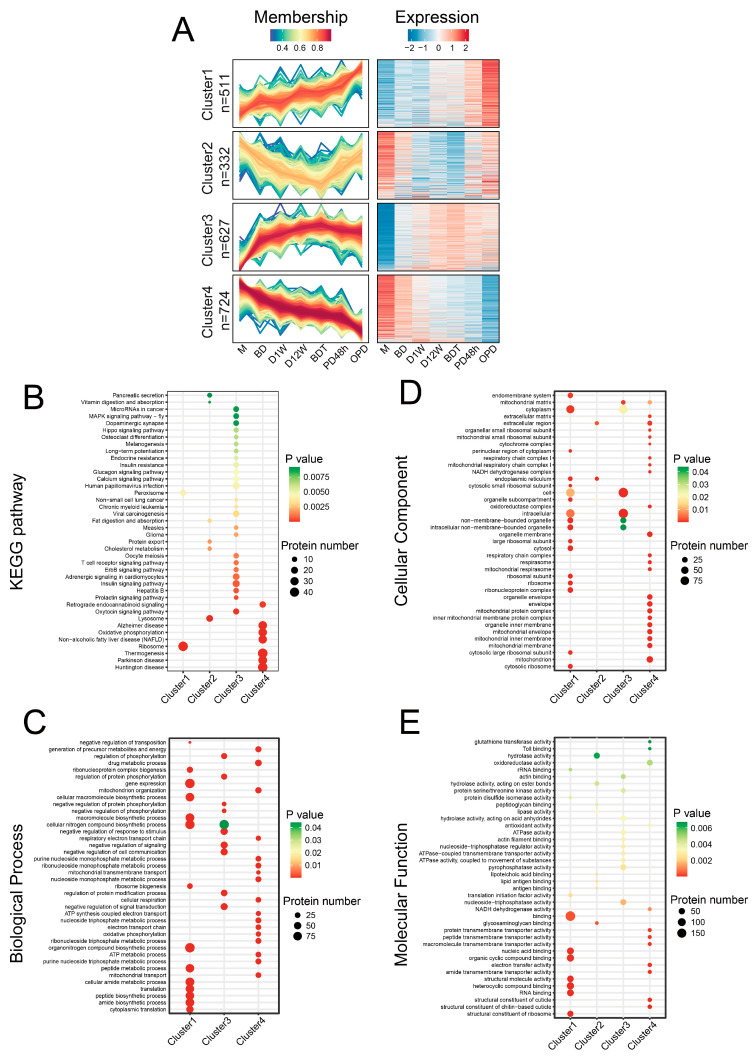
Clustering of protein expression profiles of *Bombus terrestris* using a fuzzy c-means algorithm. (**A**) Expression patterns of four clusters generated by Mfuzz software. (**B**) KEGG enrichment analysis of the four clusters. GO enrichment analysis of (**C**) molecular function, (**D**) biological process, and (**E**) cell component subcategories.

Mfuzz analysis was performed to assess the expression profiles of proteins in the seven diapause stages. The relative abundances of 4112 identified proteins were first converted to log2 logarithms to screen out significantly altered proteins, and proteins with standard deviation (SD) > 0.4 were screened. Clustering analysis was employed to divide the remaining 2194 proteins into four clusters with similar temporal expression profiles. GO/KEGG enrichment analyses (*p* < 0.05) were conducted to study the biological characteristics of proteins in different clusters (Figure 3A).

A total of 511 proteins were assigned to cluster 1, the members of which showed an increasing trend over time. KEGG and GO analyses both revealed that the prominent protein groups in cluster 1 were associated with ribosome biogenesis, protein biosynthesis, and metabolism (Figure 3).

A total of 332 proteins were assigned to cluster 2, the members of which showed a steady decrease from M to BDT but were significantly increased from BDT to OPD. The digestion-related pathway was highly represented in cluster 2, including fat and vitamin digestion and absorption, pancreatic secretion, cholesterol metabolism, protein export, and lysosomes among KEGG entries and lipase activity, hydrolase activity, and glycosaminoglycan binding among GO MF entries (Figure 3).

A total of 627 proteins were assigned to cluster 3, the members of which showed an increase from M to BDT but were slowly decreased from BDT to OPD. KEGG analysis revealed that the proteins in cluster 3 were mainly associated with the nervous and endocrine systems (dopaminergic synapse, insulin, glucagon, adrenergic, prolactin, and oxytocin), signal transduction (MAPK, Hippo, and calcium signaling pathways), and immunity and disease (T cell receptor signaling pathway, microRNAs in cancer, viral carcinogenesis, etc.; Figure 3).

A total of 724 proteins were assigned to cluster 4, the members of which showed an opposite trend to those of cluster 1.

KEGG analysis revealed that oxidative phosphorylation, non-alcoholic fatty liver disease (NAFLD) proteins, thermogenesis, and Parkinson/Huntington/Alzheimer disease entries were enriched in cluster 4. The GO BP terms ATP metabolic process and electron transport chain, CC terms oxidoreductase complex, and mitochondrial respirasome and MF terms oxidoreductase activity and electron transfer activity were highly represented among cluster 4 proteins, with all the terms being related to mitochondria (Figure 3).

### 2.4. Lipid Metabolism during Diapause

**Figure 4:**
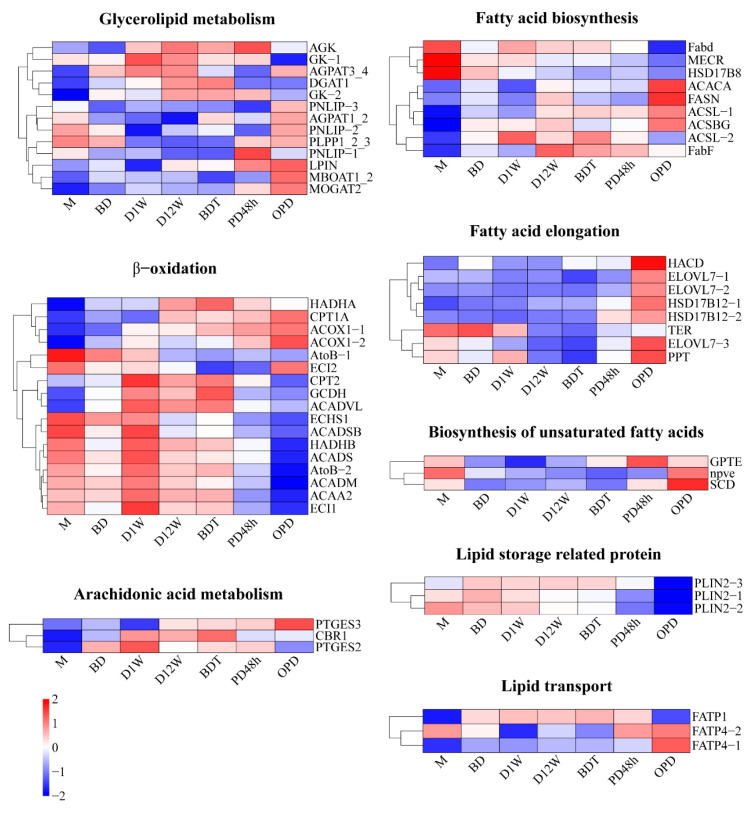
Heatmap of proteins involved in fat metabolism in *Bombus terrestris*. Results are presented as means of three independent samples. Scale bar shows upregulation (red) and downregulation (blue). The magnitude of the regulation is illustrated by the intensity of the color, and the number on the scale bar represents log2 (mean relative quantitative value).

At the diapause preparation stage of M and BD, fatty acid biosynthesis, fatty acid elongation, biosynthesis of unsaturated fatty acids, and lipid storage were the key processes. Within the diapause context (D1W, D12W, and BDT), glycerolipid metabolism, β-oxidation, and arachidonic acid metabolism were the primary processes. Within the context of post-diapause reproduction (PD48h and OPD), biosynthesis and elongation of (unsaturated) fatty acids, glycerolipid metabolism, and lipid transport were the key processes (Figure 4 and Supplementary Table S3).

The expression heatmap of proteins involved in glycerolipid metabolism showed that acylglycerol kinase (AGK), glycerol kinase (GK-1 and GK-2), 1-acyl-sn-glycerol-3-phosphate acyltransferase gamma (AGPAT3_4), and diacylglycerol O-acyltransferase 1(DGAT1) were relatively upregulated in diapause stages. All five of these proteins are related to TAG biosynthesis. Meanwhile, the proteins that were significantly upregulated during post-diapause reproduction, such as pancreatic triacylglycerol lipase (PNLIP) and 2-acylglycerol O-acyltransferase 1 (MOGAT2), were associated with both TAG biosynthesis and degradation (Figure 4 and Supplementary Table S3). These results indicate that TAG was mainly utilized at post-diapause stages.

β-oxidation is the primary pathway for fatty acids degradation and is critical for maintaining energy homeostasis, particularly in fasting situations when glucose availability is limited. Most of the proteins involved in β-oxidation have their highest expression levels at the D1W stage, such as carnitine O-palmitoyltransferase 2 (CPT2), very long-chain specific acyl-CoA dehydrogenase (ACADVL), short/branched chain specific acyl-CoA dehydrogenase (ACADSB), trifunctional enzyme subunit beta (HADHB), short-chain-specific acyl-CoA dehydrogenase (ACADS), acetyl-CoA acetyltransferase (AtoB-2), medium-chain-specific acyl-CoA dehydrogenase (ACADM), 3-ketoacyl-CoA thiolase (ACAA2), and enoyl-CoA delta isomerase 1 (ECI1) (Figure 4 and Supplementary Table S3). These results suggest that fatty acids were mainly utilized at the early diapause stage.

### 2.5. Carbohydrate Metabolism during Diapause

**Figure 5:**
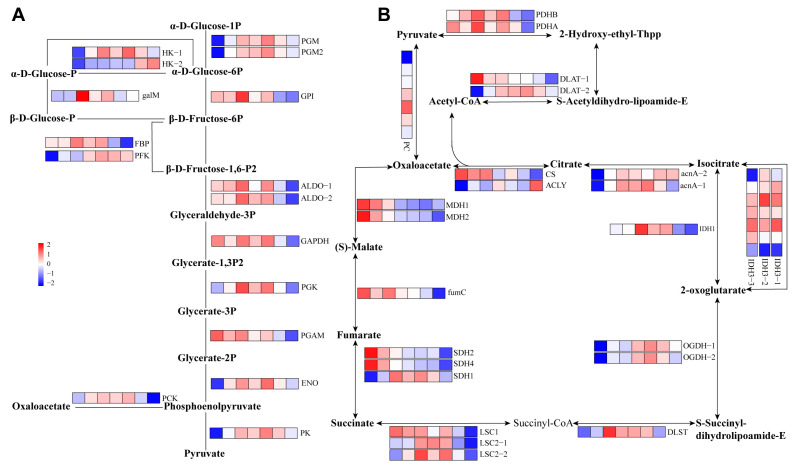
Heatmap of genes and proteins involved in glycolysis (**A**) and the TCA cycle (**B**) in *Bombus terrestris*. Results are presented as means of three independent samples. Scale bar shows upregulation (red) and downregulation (blue). The magnitude of the regulation is illustrated by the intensity of the color, and the number on the scale bar represents log2 (mean relative quantitative value).

The major proteins involved in glycolysis were considerably more abundant in D1W, D12W, and BDT compared with other non-diapause stages, including phosphoglucomutase (PGM and PGM2), glucose-6-phosphate isomerase (GPI), fructose-bisphosphate aldolase (ALDO-1 and ALDO-2), glyceraldehyde 3-phosphate dehydrogenase (GAPDH), phosphoglycerate kinase (PGK), enolase (ENO), pyruvate kinase (PK), phosphoenolpyruvate carboxykinase (PCK), fructose-1,6-bisphosphatase I (FBP), aldose 1-epimerase (GALM), and hexokinase (HK-1; Figure 5 and Supplementary Table S4). Regarding the TCA cycle, pyruvate dehydrogenase (PDHA and PDHBA), dihydrolipoyllysine-residue acetyltransferase (DLAT-2), pyruvate carboxylase (PC), aconitate hydratase (acnA-1 and acnA-2), isocitrate dehydrogenase (IDH3-1/-2/-3, IDH1), 2-oxoglutarate dehydrogenase (OGDH-1/-2), dihydrolipoyllysine-residue succinyltransferase (DLST), succinate-CoA ligase (LSC2-1/-2), and succinate dehydrogenase (SDH1) showed significantly higher expression levels in D1W, D12W, and BDT than in non-diapause stages. Moreover, dihydrolipoyllysine-residue acetyltransferase (DLAT-1), citrate synthase 2 (CS), malate dehydrogenase (MDH1and MDH2), fumarate hydratase (fumC), succinate dehydrogenase (SDH2 and SDH4), and succinate—CoA ligase (LSC1) were more abundant during pre-diapause stages (Figure 5 and Supplementary Table S4). The major proteins involved in the TCA cycle also had higher expression levels in diapause stages than in non-diapause stages. Almost all genes related to glycolysis and the TCA cycle showed lower protein abundance in the OPD stage.

### 2.6. Substance Content and Phenotypic Changes in Different Diapause Stages

*B. terrestris* were fed sugar water and pollen before M, sugar water only between stage M and BD, subjected to feeding stops between BD and D12W, and only sugar water between stage BDT and PD48h before restarting sugar water and pollen feeding.

**Figure 6:**
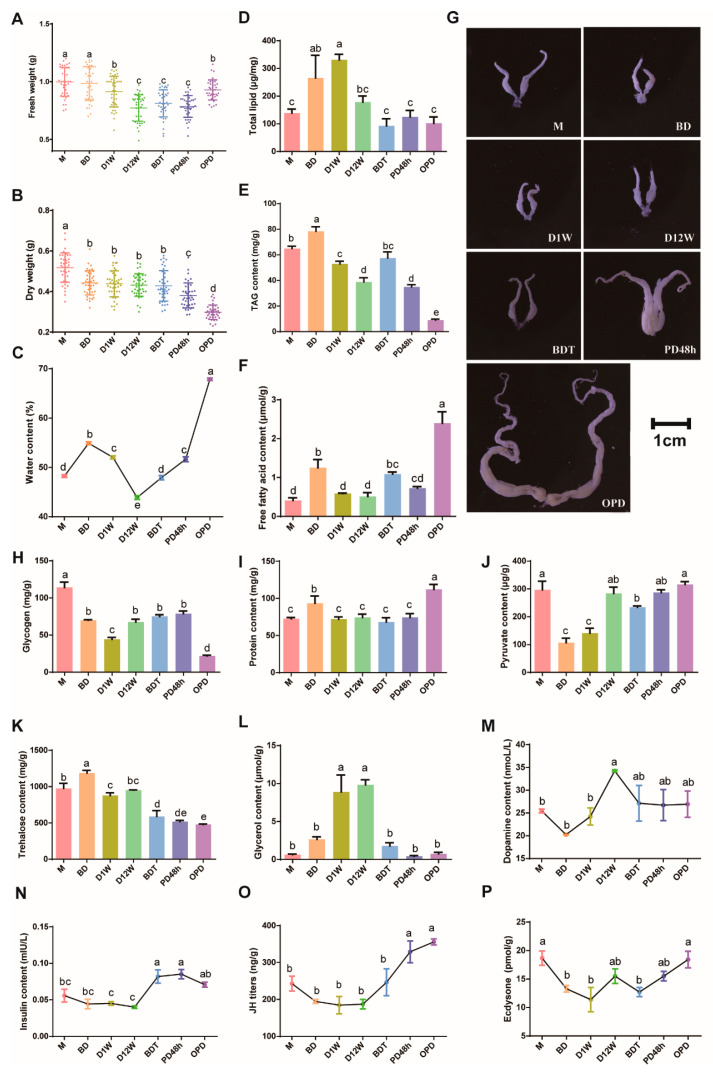
Substance content determination of *Bombus terrestris* for fresh weight (**A**), dry weight (**B**), water content (**C**), total lipids (**D**), TAG (**E**), FFAs (**F**), ovary phenotypes (**G**), glycogen (**H**), proteins (**I**), pyruvate (**J**), trehalose (**K**), glycerol (**L**), dopamine (**M**), insulin (**N**), JH titers (**O**), and ecdysone (**P**).

The fresh body weight of queens was the highest at M and BD, subsequently decreased, reached the lowest values at D12W, then slowly increased until reaching the same level as D1W during OPD (Figure 6A). This trend is essentially consistent with that of feeding time. OPD had the highest water, FFA, and protein content (Figure 6C,F,I) but the lowest dry weight and TAG content, which may be related to the development of ovaries (Figure 6B,E,G). D12W had the lowest water content and the highest glycerol content, both of which are suggested to be related to enhancing cold tolerance (Figure 6C,L). Total lipids, TAG, FFA, and protein levels were all increased at BD, which suggests the reservation of energy substances before diapause (Figure 6D–F,I). The decrease in glycogen and pyruvate content from M to D1W indicates that glycogen may be the first substance consumed during the early diapause period, while the increase from D1W to D12W indicates that lipids may be converted to glycogen for energy supply during the late diapause period (Figure 6H,J). The trehalose content was not increased continuously as glycerol after entering diapause, indicating that *B. terrestris* was not trehalose accumulating type (Figure 6K).

Insect hormones reportedly involved in diapause regulation were determined, and dopamine was abundant at D12W (Figure 6M). JH titers and insulin levels displayed the same trend as ovarian development, increasing significantly during late diapause and post-diapause periods (Figure 6N,O). JH synthesis genes mevalonate kinase (MVK) and adenosine kinase 1 (ADK-1), JH response gene Kruppel homolog 2 (Kr-h2), and JH degradation gene juvenile hormone epoxide hydrolase 1 (JHEH) were downregulated in diapause stages and upregulated in post-diapause stages (Supplementary Figure S3, Supplementary Table S5). Most genes involved in insulin signaling pathways were highly expressed in late diapause stages, such as AKT and mTOR (Supplementary Figure S3, Supplementary Table S5). The 20E content was higher at pre-diapause and post-diapause than diapause stages (Figure 6P).

## 3. Discussion

**Figure 7:**
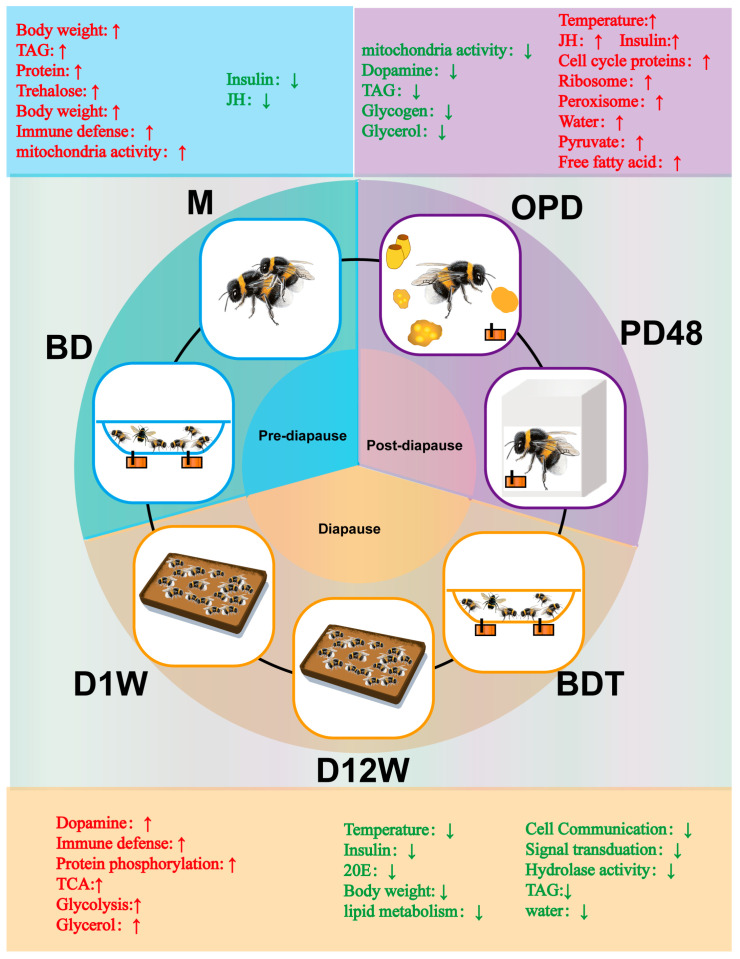
Summary of protein expression profiles and altered substances during pre-diapause, diapause, and post-diapause in *Bombus terrestris*. Red arrows represent upregulation, and the green arrows represent downregulation.

In this study, we determined the profiles of proteins related to diapause in *B. terrestris* at a larger scale than ever before and explored dynamic changes in other substances (summarized in Figure 7). We found that immune-related proteins were represented in the top 50 most abundant proteins after mating, and many immune-defense-related pathways were represented in cluster 3 of Mfuzz, which showed increased expression during the diapause phase. Colgan likewise found that immune levels were increased after mating and maintained throughout diapause [16]. This is probably because the arrest of reproductive potential that is about to undergo diapause may free up resources for other costly physiological processes, such as immunity, which can protect queen bees from infection attack and improve survival rate after mating and during diapause.

Energy metabolism involves the utilization of lipids, carbohydrates, and proteins. Energy utilization is a dynamic process during diapause, and insects appear to be capable of perceiving their energy reserves and using this information to regulate whether or not they enter diapause and for how long [19].

Many insects accumulate large amounts of lipids, proteins, and carbohydrates before diapause. In general, stored lipids, which not only provide energy for life activities but can also be converted into sugars and alcohols mutually, are the key nutrients to cope with energy shortages during diapause periods [20]. In the present study, total lipids, TAG, and FFAs accumulated during M and BD and were utilized during diapause. TAG is the main form of lipid storage and the most important energy substance in most diapause insects, and its content is closely related to the occurrence, maintenance, and termination of diapause [21,22]. *Mamestra brassicae* and *Chrysoperla nipponensis* reserve lipids before diapause [22,23]. Lipid reserves remaining after termination of diapause are readily used for subsequent egg production [19], which was also observed in the mosquito *Culex pipiens* [24]. These results suggest that lipids are mainly used as energy substances for storage to ensure that *B. terrestris* queens have enough energy to survive diapause in a harsh environment. Meanwhile, TAG decreased dramatically during the ovipositing period, indicating that it plays an important role in the very energy-intensive process of ovarian development and accumulation of vitellogenin in ovaries.

Catabolism of sugar occurs during glycolysis and subsequent pyruvate oxidation, and products are used in the citric acid (TCA) cycle to generate substrates for oxidative phosphorylation [25]. Glycogen is a major energy substance in insects. Trehalose, the main hemolymph sugar in insects, serves as an energy source and protectant against severe environmental stresses, including freezing, starvation, and oxidation [26,27].

Compared with pre-diapause, most of the proteins involved in glycolysis and the TCA pathway were upregulated during diapause, accompanied by a decrease in glycogen and trehalose content in this study. Pyruvate, the most important intermediate metabolite among energy substances, exhibited the same trend as glycogen from M to PD 48 h periods. Trehalose accumulated at BD, while glycogen content decreased significantly faster than trehalose content during diapause, and trehalose did not change much in the later period (D1W to D12W). The rapid decrease in glycogen content in the initial stage of diapause was also observed in *Sericinus montelus* and *Hyphantria cunea* pupae. However, unlike the trehalose that increased first and then decreased in *B. terrestris*, the trehalose levels in *S. montelus* and *H. cunea* remained high during the diapause maintenance period [28,29]. These results suggest that glycogen acts as a caloric reserve during early diapause and shifts to depend on other energy stores in the middle and late diapause stages. Furthermore, it is not trehalose but glycerol that has a high level during the diapause period and acts as the main low-temperature protective agent, while trehalose may act as a protective agent in a short period of time in the early diapause stage and interconvert with glycogen to participate in diapause regulation in *B. terrestris*.

In general, insects accumulate significant amounts of protein during the diapause preparation period, possibly because it can improve cold tolerance. It has been reported that insects accumulate amino acids in specific proteins, and different insects reserve different kinds of amino acids according to their needs to form antifreeze proteins or antifreeze peptides so as to improve the cold resistance during diapause. These proteins are synthesized before diapause, and their component amino acids can be used for intermediate metabolism and respiratory metabolism during diapause, as well as for the regrowth and development at post-diapause [30,31]. With diapause development, the protein content was significantly decreased, which indicated that the protein accumulated in the pre-diapause period may be degraded into amino acids to participate in metabolism, or act as a low-temperature protective agent to alleviate cold damage. During post-diapause, protein content increased. The protein content increase during post-diapause may be due to protein being the main structural substance. Furthermore, reproductive system development is stagnated during diapause, especially ovarian development and vitellogenin synthesis. Meanwhile, during the OPD stage, *B. terrestris* vitellogenin (LOC100650436) increased by about 50 times compared with the diapause stage. The protein content of reproductive diapause individuals of the *Colaphellus bowringi* was also significantly lower than that of non-reproductive individuals [32]. These results suggest that protein, as an important structural substance and biological function in insects, is closely related to diapause induction, maintenance, termination, and oviposition. KEGG pathway and GO enrichment analysis of the Mfuzz cluster 1 proteins showed that proteins involved in ribosome, translation, amide biosynthetic process, peptide biosynthetic process, peptide metabolic process, organonitrogen compound biosynthetic process, macromolecular biosynthetic process, gene expression, and ribonucleoprotein complex biogenesis were significantly represented, which has an increasing trend from M to OPD. These results suggest that proteins involved in protein biogenesis and metabolism-related pathways play an important role during the diapause of *B. terrestris* queens.

Oxidative phosphorylation, the main metabolic pathway for releasing chemical energy in eukaryotes, generates ATP in the inner membrane of mitochondria. Herein, most of the genes involved in oxidative phosphorylation showed a gradually decreasing trend from the M to OPD stages. Mitochondria-related functional proteins such as cytochrome c oxidase subunit (LOC100642567/LOC100651780/LOC100643317/LOC100647597/), NADH dehydrogenase 1 alpha/beta subcomplex subunit (LOC100647452/LOC100646263/LOC105665884/LOC100649271), and cytochrome b-c1 complex subunit (LOC100646316/LOC100644237/LOC100644605) were downregulated from M to OPD, which suggests that mitochondria may be dysfunctional during diapause. Metabolic depression is heightened by inhibitions within mitochondria during diapause [33]. However, the sustained reduction in oxidative phosphorylation in the post-diapause phase compared to the diapause phase seems difficult to understand, but the same results have also been reported in our previous study, which may require further investigation [10].

In the present study, superoxide dismutase (SOD) (LOC100646662\LOC105666244\LOC100642760), peroxidase (POD) (LOC100646227\LOC105666558\LOC100644635\LOC100644139\LOC100644975\LOC100648954), glutathione peroxidase (GSH-Px) (LOC100642912\LOC100648859), and glutathione-S-transferase (GST) (LOC100651035\LOC100650722\LOC100650878\LOC100651606\LOC100646009\LOC100652331) protein abundance in mitochondria during diapause were reduced compared with the pre-diapause stage, which had the same trend as oxidative phosphorylation (Supplementary Table S1). Reactive oxygen species (ROS) are also produced alongside ATP via the electron transport chain during oxidative phosphorylation [34]. ROS formation can induce oxidative stress, leading to cell damage and eventually cell death. Antioxidant enzymes such as SOD, POD, GST, and GSH-Px can alleviate the destructive effects of ROS and delay cell aging [35]. Together, these results imply a low metabolic rate of mitochondria and low levels of ROS accumulation in *B. terrestris*, which do not feed and are practically immobile during diapause.

Many studies have reported that insect hormones are pivotal regulators in diapause induction, maintenance, and termination [36,37,38,39]. Most early research on adult diapause hormonal control focused on JH. Diapause-destined *Leptinotarsa decemlineata* has a low JH titer after adult emergence, remains low during diapause, and increases following diapause termination [40]. JH application could terminate diapause in *C. pipiens*, as shown by reversing the diapause-like arrest caused by knockdown of two ribosomal genes [41,42]. In this study, JH remained at a low level at pre-diapause and diapause periods and increased significantly after diapause termination. Despite strong evidence linking JH to reproductive diapause, JH is not the sole factor related to diapause induction, maintenance, and termination. Diapausing females did not develop massive fat deposits after being injected with dsRNA against forkhead transcription factor (FOXO), a gene downstream of insulin. It was presumed to be that the interruption of insulin signaling inhibits JH synthesis and activates FOXO, resulting in the diapause phenotype [30]. The similar trend of insulin and JH in *B. terrestris* suggests that insulin may play a regulatory role in the upstream of JH during diapause. Both JH and 20E are the two common hormones that regulate Vg synthesis and may play different roles in different insects. For example, JH regulates Vg synthesis in fat bodies of *Tribolium castaneum*, 20E is involved in ovarian development and oocyte maturation, and synthesis of Vg in *Bombyx mori* and *Spodoptera frugiperda* is mainly regulated by 20E [43,44,45,46]. Moreover, 20E stimulates endocytosis of YPs in a dose-dependent manner at diapause termination to start vitellogenesis in *Drosophila melanogaster* [47,48]. JH and insulin titers showed the same trends as ovary development, while 20E showed no significant relevant change. These results suggest that JH may play a more important role than 20E in post-diapause ovarian development in *B. terrestris*. Our proteomic analysis showed that dopaminergic synapses were enriched in cluster 3 in Mufzz analysis, and related proteins were upregulated in diapause stages. Dopamine content was also elevated in diapause stages. Dopamine is the key factor for the onset of egg diapause in silkworm *B. mori* and pupal diapause in cabbage armyworm *Mamestra brassicae* [49,50]. Dopamine modulates temperature sensitivity; low dopamine levels prefer higher temperatures and vice versa [50,51]. Dopamine also functions as a negative regulator of glucose-stimulated insulin secretion [52]. The high level of dopamine at D12W may contribute to cold temperature tolerance. Meanwhile, insulin content was low in diapause stages, which may indicate a negative regulator function of dopamine on insulin.

## 4. Materials and Methods

### 4.1. Sample Preparation

Queens of *B. terrestris* were generated at Shandong Lubao Technology Co. Ltd. (Jinan, China), which specializes in the commercial production of bumblebees, and thus has extensive expertise and facilities to create sample groups using standard commercial practices. Queens were collected at seven different development stages: (I) mated, (II) before diapause, (III) diapause for one week, (IV) diapause for 12 weeks, (V) before diapause termination, (VI) post-diapause 48 h, and (VII) oviposition post-diapause. Bees were frozen in liquid nitrogen and stored at −80 °C until use. Each group included three biological replicates and each replicate included three queen bees.

Mated (M): At five to seven days of age, virgin queens were grouped with unrelated males and placed in the mating cage until mating took place. Mating couples remained connected for 15−30 min and mated queens were therefore sampled immediately post-mating.

Before diapause (BD): Mated queens were placed in the dark at two different low temperatures for two days. By the end of this transition stage, queens were collected and placed in liquid nitrogen.

Diapause for one week and diapause for 12 weeks (D1W and D12W): Queens that had passed through the transition stage were kept in the dark for 1 week or 12 weeks at 4 °C and samples were collected. Prior to sampling, signs of life (abdominal movements or leg twitching) were searched for to confirm queens had survived diapause.

Before diapause termination (BDT): After 12 weeks of diapause, two different temperature stages of transition and adaptation were investigated. CO_2_ treatment was required for the next step, and samples were collected prior to CO_2_ treatment.

Post-diapause 48 h (PD48h): Diapause queens were treated with CO_2_ for 1 min, and this was repeated after 24 h at room temperature. Queens were then fed 50% sucrose for two days at 28 °C and sampled.

Post-diapause oviposition (OPD): Post-diapause queens were kept at 28 °C and sampled after egg-laying was observed.

### 4.2. Protein Extraction and Trypsin Enzymatic Digestion

Samples in each group were fully ground to powder with liquid nitrogen, four-fold volume of phenol extraction buffer containing 10 mM Dithiothreitol (DTT), and 1% protease inhibitor cocktail was added, and ultrasonic treatment was performed. An equal volume of Tris-phenol was added, and samples were centrifuged at 5500× *g* for 10 min at 4 °C. The supernatant was mixed with five-fold volume of 0.1 M ammonium acetate/methanol and left to precipitate overnight. Precipitate was washed with methanol then with acetone, resuspended in 8 M urea, and the protein concentration was determined with a BCA kit (Thermo Fisher Scientific, Waltham, MA, USA) according to the manufacturer’s instructions. For digestion, proteins in each sample were enzymatically hydrolyzed in equal quantities, and the volume was adjusted to be consistent with the lysate. TCA was slowly added to a final concentration of 20% with vortex mixing and samples were precipitated for 2 h at 4 °C. Centrifugation was performed at 4500× *g* for 5 min, the supernatant was discarded, and the pellet was washed with pre-cooled acetone for two or three times. After drying, TEAB was added to a final concentration of 200 mM, the pellet was lysed by ultrasound, trypsin was added at a 1:50 ratio of protease: protein (m/m), and samples were digested overnight. DTT was added to a final concentration of 5 mM and samples were reduced for 30 min at 56 °C. Iodine acetamide (IAA) was added to a final concentration of 11 mM and samples were incubated for 15 min at room temperature away from light.

### 4.3. 4D-LFQ LC–MS Analysis

Tryptic peptides were dissolved in solvent A (0.1% formic acid and 2% acetonitrile in water) and directly loaded onto a home-made reversed-phase analytical column (25 cm length, 100 μm internal diameter). Peptides were separated with a gradient from 6% to 23% solvent B (0.1% formic acid in 90% acetonitrile) over 68 min, 23% to 32% in 14 min, and increasing to 80% over 4 min and holding at 80% for 4 min, all at a constant flowrate of 500 nL/min on an EASY-nLC 1200 UPLC system (Thermo Fisher Scientific).

The separated peptides were analyzed using an Exploris 480TM instrument (Thermo Fisher Scientific) equipped with a nano-electrospray ion source. The electrospray voltage applied was 2.3 kV and the compensation voltage was −45 V or −65 V. The full MS scan resolution was set to 60,000 for a scan range of 400–1200 *m*/*z*. The 25 most abundant precursors were then selected for further MS/MS analyses with 20 s dynamic exclusion. HCD fragmentation was performed at a normalized collision energy (NCE) of 27%. Fragments were detected in the Orbitrap at a resolution of 15,000. Fixed first mass was set as 110 *m*/*z*, automatic gain control (AGC) target was set at 100% with an intensity threshold of 5 × 10^4^ ions/s, and MS2 maximum injection time was set as Auto.

### 4.4. The Proteome Database Searching

Secondary MS data were retrieved by Proteome Discoverer (V2.4.1.15). Proteins were detected in Blast_Bombus_terrestris_30195_PR_20201214.fasta (17,032 sequences) using the common contamination database. The additional reverse database was added to calculate false discovery rate (FDR) caused by random matching. The enzyme digestion mode was set to Trypsin (Full), the number of missing sites was set to two, the minimum length of peptides was set to six amino acid residues, the maximum modification number of peptides was set to three, the mass error tolerance of primary parent ions was set to 10 ppm, and the mass error tolerance of secondary fragment ions was set to 0.02 Da. Carbamidomethyl (C) was set as a fixed modification, and Oxidation (M), Acetyl (N-terminus), Met-loss (M), and Met-Loss + Acetyl (M) were set as variable modifications. FDRs for proteins, peptides, and PSM identification were all set at 1%. In order to obtain high-quality analysis results, database search analysis results were subject to further data filtering, and identified proteins must include at least one unique peptide.

### 4.5. Bioinformatics and Annotations

Proteins with fold-change ratio ≥ 1.5 or ≤0.67 (*p* < 0.05) were considered significantly differentially abundant. The fold change calculation formula is shown as following: R denotes the relative quantitative value of the protein, i denotes the sample, and k denotes the protein. FC_A/B,k_ = Mean(R_ik_, i∈A)/Mean(R_ik_, i∈B). To calculate the statistical significance of difference between groups, the Student’s *t*-test was performed on the relative quantitative value of each protein from the two sample groups. *p* value < 0.05 was usually considered as the threshold for significance. Therefore, the relative quantitative value of proteins was applied with log2 transformation typically. The formula is shown as following: Pik = T.test(Log2(Rik, i∈A), Log2(Rik, i∈B)).

Gene Ontology (GO) annotation and enrichment: Eggnog-mapper software (version 2.0) based on the EggNOG database was used to conduct GO annotation, which can be divided into three categories: Biological process (BP), Cellular Component (CC), and Molecular Function (MF). DAPs were enriched against all identified proteins by two-tailed Fisher’s exact test (*p* < 0.05).

Subcellular localization analysis: WoLF PSORT (version 1.0; https://wolfpsort.hgc.jp/, accessed on 15 March 2022) was used to predict subcellular localization of the proteins.

Kyoto Encyclopedia of Genes and Genomes (KEGG) annotation and enrichment: Pathway enrichment was derived from the KEGG database (version 2.5; https://www.kegg.jp/kegg/pathway, accessed on 20 March 2022) by two-tailed Fisher’s exact test (*p* < 0.05).

The KOG database (Eukaryotic Orthologous Groups) (version 2003; https://ftp.ncbi.nih.gov/pub/COG/KOG/, accessed on 21 March 2022) was employed for functional annotation.

Domain and enrichment analysis: The Pfam database and the corresponding PfamScan tool were used to annotate the domains of identified proteins, and a two-tailed Fisher’s exact test (*p* < 0.05) was applied to assess enrichment of DAPs.

The R package Mfuzz was used to analyze proteins with similar expression patterns.

Heatmap analysis: The mean relative quantitative value of proteins was applied with log2 transformation, and log2 (mean relative quantitative value) was used for Advanced Heatmap barplot analysis using the OmicStudio tools at http://www.omicstudio.cn/tool, accessed on 28 May 2023.

### 4.6. Measurement of Weight, Water, Total Lipids, Triacylglycerol (TAG), and Free Acid Content

The fresh weight (FW) of *B. terrestris* adult queens was measured directly by an electronic microbalance (n ≥ 30). Dry weight (DW) was measured after drying in an oven for 72 h at 60 °C (n ≥ 30). Dry adults were ground into powder in liquid nitrogen, then transferred into a new Eppendorf tube. Chloroform/methanol (2:1) was added at a ratio of 1 g to 10 mL, samples were vortexed thoroughly, then centrifuged at 2600× *g* for 10 min, and the supernatant was removed. Extraction and centrifugation steps were repeated and pellets were dried in an oven for 72 h at 65 °C. Dried pellets (DPs) were weighted by using an electronic microbalance (n ≥ 10). Water content was calculated using the formula (FW − DW)/FW × 100%, and total lipid content was calculated using the formula (DW − DP)/FW [53]. TAG content and free fatty acid (FFA) content were measured using a TAG content assay kit (Solarbio, Beijing, China) and free fatty acids (FFA) content assay kit (Solarbio), respectively.

### 4.7. Measurement of Other Substances

Dopamine and juvenile hormone (JH) were determined by enzyme-linked immunosorbent assay (Abmart, Shanghai, China). Insulin and ecdysone were determined by enzyme-linked immunosorbent assay (Mlbio, Shanghai, China). Glycogen, pyruvate, and trehalose content were measured using glycogen, pyruvate, and trehalose content assay kits (Solarbio), respectively. Glycerol content was measured using a glycerol assay kit (Nanjing Jiancheng Bioengineering Institute, Nanjing, China). Protein content was determined by BCA protein quantification assay (Biorun, Wuhan, China). Three biological replicates were included for each measurement.

### 4.8. Statistical Analysis

One-way analysis of variance (ANOVA) was used to determine statistical differences among samples, followed by separation of means using Tukey’s test, and differences were considered significant at *p* < 0.05. All data were analyzed by SPSS software (version 26.0).

## 5. Conclusions

This study provides a model of dynamic changes in proteins and other substances during pre-diapause, diapause, and post-diapause. Regulation of *B. terrestris* diapause is strongly associated with insect hormones, energy storage and utilization, cold tolerance substances, and signal transduction pathways. The transition from pre-diapause to diapause is highlighted by an increase in dopamine content, immune defense, and protein phosphorylation and a decrease in content of insulin and 20E, lipid metabolism, and cell communication. The termination of diapause is mainly highlighted by an increase in JH, insulin, cell cycle proteins, and free fatty acid and a decrease in dopamine, TAG, glycogen, and glycerol. These results provide greater understanding regarding the molecular mechanisms and substance basis underlying diapause in bumblebees.

## Data Availability

The mass spectrometry proteomics data have been deposited to the ProteomeXchange Consortium (http://proteomecentral.proteomexchange.org, accessed on 20 November 2023) via the iProX partner repository [54,55] with the dataset identifier PXD047146.

## Supplementary Materials

The following supporting information can be downloaded at https://www.mdpi.com/article/10.3390/ijms25010326/s1.

## References

[1] MacRae T.H. (2010). Gene expression, metabolic regulation and stress tolerance during diapause. Cell. Mol. Life Sci..

[2] Amsalem E., Galbraith D.A., Cnaani J., Teal P.E.A., Grozinger C.M. (2015). Conservation and modification of genetic and physiological toolkits underpinning diapause in bumble bee queens. Mol. Ecol..

[3] Taylor F. (1980). Optimal switching to diapause in relation to the onset of winter. Theor. Popul. Biol..

[4] Denlinger D.L. (2002). Regulation of diapause. Annu. Rev. Entomol..

[5] Koštál V. (2006). Eco-physiological phases of insect diapause. J. Insect Physiol..

[6] Denlinger D.L. (1981). Basis fro a skewed sex ratio in diapause-destined flesh flies. Evolution.

[7] Irwin J.T., Lee R.E. (2000). Mild winter temperatures reduce survival and potential fecundity of the goldenrod gall fly, *Eurosta solidaginis* (Diptera: Tephritidae). J. Insect Physiol..

[8] Velthuis H.H.W., Duchateau M.J. (1988). Development and reproductive strategies in *Bombus terrestris* colonies. Behaviour.

[9] Velthuis H.H.W., van Doorn A. (2006). A century of advances in bumblebee domestication and the economic and environmental aspects of its commercialization for pollination. Apidologie.

[10] Liu Y., Wang R., Su L., Zhao S., Dai X., Chen H., Wu G.A., Zhou H., Zheng L., Zhai Y. (2022). Integrative Proteomic and Phosphoproteomic Analyses Revealed Complex Mechanisms Underlying Reproductive Diapause in *Bombus terrestris* Queens. Insects.

[11] Chen H., Wu G.A., Zhou H., Dai X., Steeghs N.W.F., Dong X., Zheng L., Zhai Y. (2021). Hormonal Regulation of Reproductive Diapause That Occurs in the Year-Round Mass Rearing of *Bombus terrestris* Queens. J. Proteome Res..

[12] Feder M.E., Walser J.C. (2005). The biological limitations of transcriptomics in elucidating stress and stress responses. J. Evol. Biol..

[13] Ren X.Y., Zhang L.S., Han Y.H., An T., Liu Y., Li Y.Y., Chen H.Y. (2015). Proteomic research on diapause-related proteins in the female ladybird, *Coccinella septempunctata* L.. Bull. Entomol. Res..

[14] Ma H.-Y., Zhou X.-R., Tan Y., Pang B.-P. (2019). Proteomic analysis of adult *Galeruca daurica* (Coleoptera: Chrysomelidae) at different stages during summer diapause. Comp. Biochem. Physiol. Part D Genom. Proteom..

[15] Zhai Y., Dong X., Gao H., Chen H., Yang P., Li P., Yin Z., Zheng L., Yu Y. (2019). Quantitative Proteomic and Transcriptomic Analyses of Metabolic Regulation of Adult Reproductive Diapause in *Drosophila suzukii* (Diptera: Drosophilidae) Females. Front. Physiol..

[16] Colgan T.J., Finlay S., Brown M.J.F., Carolan J.C. (2019). Mating precedes selective immune priming which is maintained throughout bumblebee queen diapause. BMC Genom..

[17] Meier F., Brunner A.D., Koch S., Koch H., Lubeck M., Krause M., Goedecke N., Decker J., Kosinski T., Park M.A. (2018). Online Parallel Accumulation-Serial Fragmentation (PASEF) with a Novel Trapped Ion Mobility Mass Spectrometer. Mol. Cell. Proteom..

[18] Brzhozovskiy A., Kononikhin A., Bugrova A.E., Kovalev G.I., Schmit P.-O., Kruppa G., Nikolaev E.N., Borchers C.H. (2022). The Parallel Reaction Monitoring-Parallel Accumulation–Serial Fragmentation (prm-PASEF) Approach for Multiplexed Absolute Quantitation of Proteins in Human Plasma. Anal. Chem..

[19] Hahn D.A., Denlinger D.L. (2011). Energetics of Insect Diapause. Annu. Rev. Entomol..

[20] Arrese E.L., Soulages J.L. (2010). Insect Fat Body: Energy, Metabolism, and Regulation. Annu. Rev. Entomol..

[21] Sinclair B.J., Marshall K.E., Suarez R.K., Hoppeler H.H. (2018). The many roles of fats in overwintering insects. J. Exp. Biol..

[22] Chen Z.Z., Wang X., Kong X., Zhao Y.M., Xu M.H., Gao Y.Q., Huang H.Y., Liu F.H., Wang S., Xu Y.Y. (2023). Quantitative transcriptomic and proteomic analyses reveal the potential maintenance mechanism of female adult reproductive diapause in *Chrysoperla nipponensis*. Pest Manag. Sci..

[23] Ding L., Li Y., Goto M. (2003). Physiological and biochemical changes in summer and winter diapause and non-diapause pupae of the cabbage armyworm, *Mamestra brassicae* L. during long-term cold acclimation. J. Insect Physiol..

[24] Zhou G., Miesfeld R.L. (2009). Energy metabolism during diapause in *Culex pipiens* mosquitoes. J. Insect Physiol..

[25] Nolfi-Donegan D., Braganza A., Shiva S. (2020). Mitochondrial electron transport chain: Oxidative phosphorylation, oxidant production, and methods of measurement. Redox Biol..

[26] Thompson S.N. (2003). Trehalose—The Insect ‘Blood’ Sugar. Adv. Insect Physiol..

[27] Elbein A.D., Pan Y.T., Pastuszak I., Carroll D. (2003). New insights on trehalose: A multifunctional molecule. Glycobiology.

[28] Zhao L., Wang X., Liu Z., Torson A.S. (2022). Energy Consumption and Cold Hardiness of Diapausing Fall Webworm Pupae. Insects.

[29] Xiao Q.H., He Z., Wu R.W., Zhu D.H. (2022). Physiological and biochemical differences in diapause and non-diapause pupae of *Sericinus montelus* (Lepidoptera: Papilionidae). Front. Physiol..

[30] Denlinger D.L., Yocum G.D., Rinehart J.P., Gilbert L.I., Iatrou K., Gill S. (2012). Hormonal control of diapause. Insect Endocrinology.

[31] Morgan T.D., Chippendale G.M. (1983). Free amino acids of the haemolymph of the southwestern corn borer and the European corn borer in relation to their diapause. J. Insect Physiol..

[32] Tan Q.Q., Feng L., Liu W., Zhu L., Lei C.L., Wang X.P. (2016). Differences in the pre-diapause and pre-oviposition accumulation of critical nutrients in adult females of the beetle *Colaphellus bowringi*. Entomol. Exp. Appl..

[33] Patil Y.N., Gnaiger E., Landry A.P., Leno Z.J., Hand S.C. (2023). OXPHOS capacity is diminished and the phosphorylation system inhibited during diapause in an extremophile, embryos of *Artemia franciscana*. J. Exp. Biol..

[34] Shadel G.S., Horvath T.L. (2015). Mitochondrial ROS Signaling in Organismal Homeostasis. Cell.

[35] Poljsak B., Šuput D., Milisav I. (2013). Achieving the Balance between ROS and Antioxidants: When to Use the Synthetic Antioxidants. Oxidative Med. Cell. Longev..

[36] Palli S.R., Guo S., Tian Z., Wu Q.-W., King-Jones K., Liu W., Zhu F., Wang X.-P. (2021). Steroid hormone ecdysone deficiency stimulates preparation for photoperiodic reproductive diapause. PLoS Genet..

[37] Sim C., Denlinger D.L. (2013). Insulin signaling and the regulation of insect diapause. Front. Physiol..

[38] Li Y.Y., Chen J.J., Liu M.Y., He W.W., Reynolds J.A., Wang Y.N., Wang M.Q., Zhang L.S. (2022). Enhanced Degradation of Juvenile Hormone Promotes Reproductive Diapause in the Predatory Ladybeetle *Coccinella Septempunctata*. Front. Physiol..

[39] Mukai A., Mano G., Des Marteaux L., Shinada T., Goto S.G. (2022). Juvenile hormone as a causal factor for maternal regulation of diapause in a wasp. Insect Biochem. Mol. Biol..

[40] de Kort C.A.D. (1990). Thirty-five years of diapause research with the Colorado potato beetle. Entomol. Exp. Appl..

[41] Kim M., Denlinger D.L. (2010). A potential role for ribosomal protein S2 in the gene network regulating reproductive diapause in the mosquito *Culex pipiens*. J. Comp. Physiol. B.

[42] Kim M., Sim C., Denlinger D.L. (2010). RNA interference directed against ribosomal protein S3a suggests a link between this gene and arrested ovarian development during adult diapause in *Culex pipiens*. Insect Mol. Biol..

[43] Swevers L., Iatrou K. (2003). The ecdysone regulatory cascade and ovarian development in lepidopteran insects: Insights from the silkmoth paradigm. Insect Biochem. Mol. Biol..

[44] Telfer W.H. (2009). Egg formation in lepidoptera. J. Insect Sci..

[45] Parthasarathy R., Sheng Z., Sun Z., Palli S.R. (2010). Ecdysteroid regulation of ovarian growth and oocyte maturation in the red flour beetle, *Tribolium castaneum*. Insect Biochem. Mol. Biol..

[46] Sheng Z., Xu J., Bai H., Zhu F., Palli S.R. (2011). Juvenile Hormone Regulates Vitellogenin Gene Expression through Insulin-like Peptide Signaling Pathway in the Red Flour Beetle, *Tribolium castaneum*. J. Biol. Chem..

[47] Gilbert L.I., Serafin R.B., Watkins N.L., Richard D.S. (1998). Ecdysteroids regulate yolk protein uptake by *Drosophila melanogaster* oocytes. J. Insect Physiol..

[48] Richard D.S., Jones J.M., Barbarito M.R., Cerula S., Detweiler J.P., Fisher S.J., Brannigan D.M., Scheswohl D.M. (2001). Vitellogenesis in diapausing and mutant *Drosophila melanogaster*: Further evidence for the relative roles of ecdysteroids and juvenile hormones. J. Insect Physiol..

[49] Noguchi H., Hayakawa Y. (2001). Dopamine is a key factor for the induction of egg diapause of the silkworm, *Bombyx mori*. Eur. J. Biochem..

[50] Noguchi H., Hayakawa Y. (1997). Role of dopamine at the onset of pupal diapause in the cabbage armyworm *Mamestra brassicae*. FEBS Lett..

[51] Jakšić A.M., Karner J., Nolte V., Hsu S.K., Barghi N., Mallard F., Otte K.A., Svečnjak L., Senti K.A., Schlötterer C. (2020). Neuronal Function and Dopamine Signaling Evolve at High Temperature in Drosophila. Mol. Biol. Evol..

[52] Uefune F., Aonishi T., Kitaguchi T., Takahashi H., Seino S., Sakano D., Kume S. (2022). Dopamine Negatively Regulates Insulin Secretion Through Activation of D1-D2 Receptor Heteromer. Diabetes.

[53] Colinet H., Vernon P., Hance T. (2007). Does thermal-related plasticity in size and fat reserves influence supercooling abilities and cold-tolerance in *Aphidius colemani* (Hymenoptera: Aphidiinae) mummies?. J. Therm. Biol..

[54] Ma J., Chen T., Wu S., Yang C., Bai M., Shu K., Li K., Zhang G., Jin Z., He F. (2019). iProX: An integrated proteome resource. Nucleic Acids Res..

[55] Chen T., Ma J., Liu Y., Chen Z., Xiao N., Lu Y., Fu Y., Yang C., Li M., Wu S. (2022). iProX in 2021: Connecting proteomics data sharing with big data. Nucleic Acids Res..

