# Supplementary material for: Comparative 4D Label-Free Quantitative Proteomic Analysis of Bombus terrestris Provides Insights into Proteins and Processes Associated with Diapause
Source: Int J Mol Sci. 2023 Dec 26;25(1):326. doi: 10.3390/ijms25010326 (PMC10778897; doi:10.3390/ijms25010326)

A

Total spectrum	Matched spectrum	Peptides	Unique peptides	Identified proteins	Quantifiable proteins
2284234	816491(35.7%)	35587	32610	4112	3519

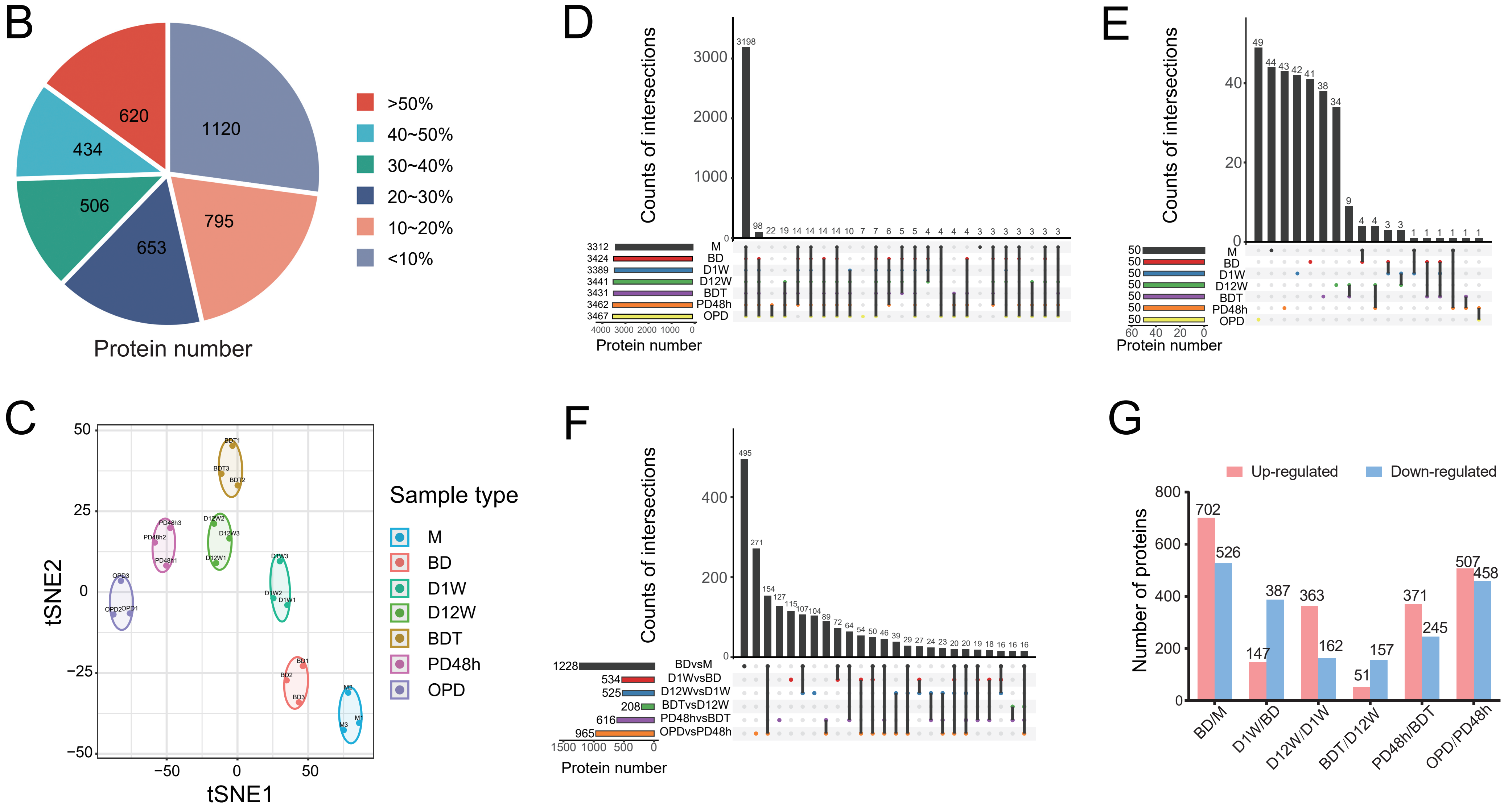

Supplement: Supplementary file 1 [file ijms-25-00326-s001.zip › Revised figures and supplementary materials/revised figures/Figure 1.pdf]
