# Supplementary material for: Comparative 4D Label-Free Quantitative Proteomic Analysis of Bombus terrestris Provides Insights into Proteins and Processes Associated with Diapause
Source: Int J Mol Sci. 2023 Dec 26;25(1):326. doi: 10.3390/ijms25010326 (PMC10778897; doi:10.3390/ijms25010326)
Supplement: Supplementary file 1 [file ijms-25-00326-s001.zip › Revised figures and supplementary materials/revised figures/Figure 4.pdf]

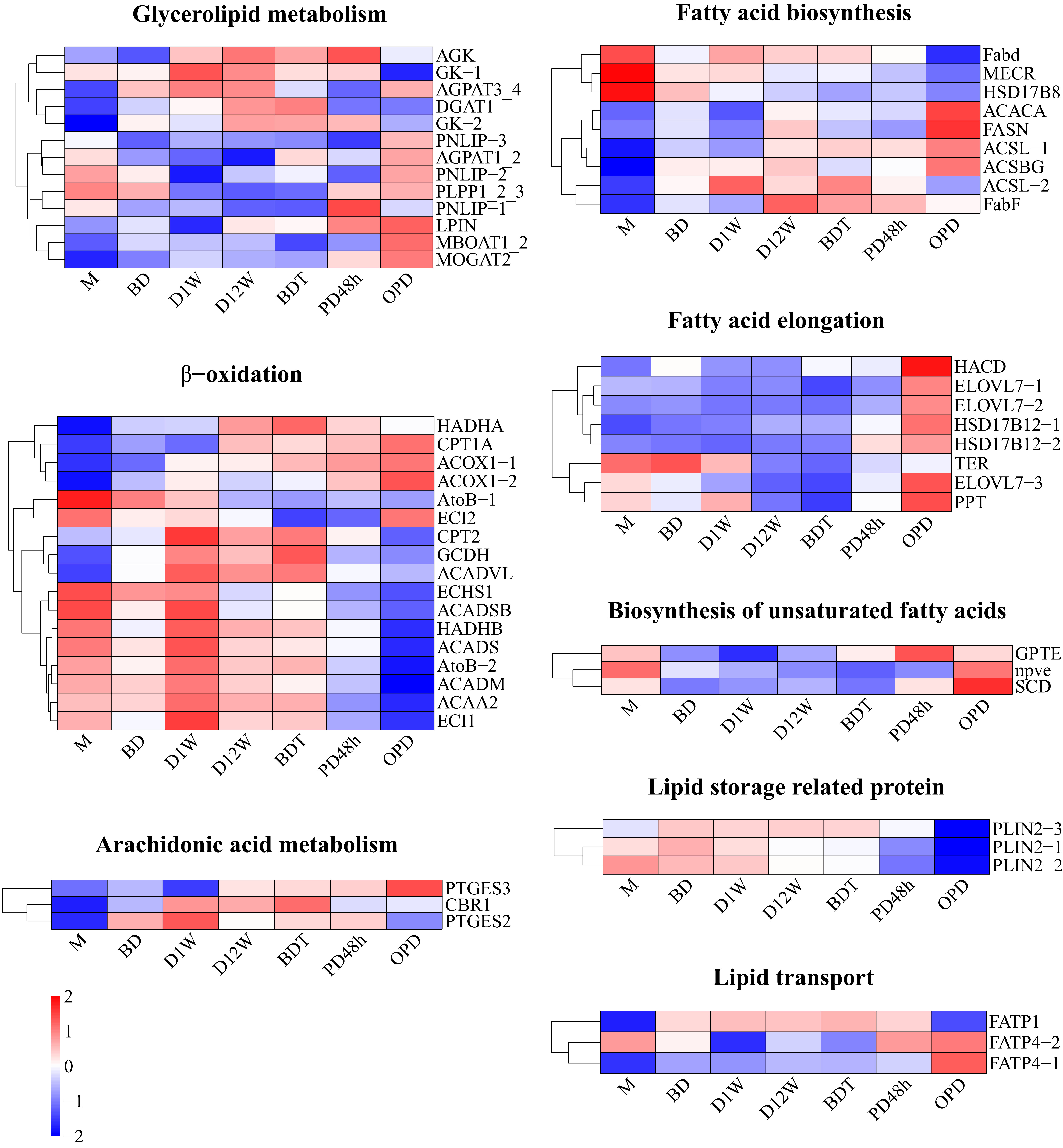
Glycerolipid metabolism
AGK
GK−1
AGPAT3_4
DGAT1
GK−2
PNLIP−3
AGPAT1_2
PNLIP−2
PLPP1_2_3
PNLIP−1
LPIN
MBOAT1_2
MOGAT2
M
BD
D1W
D12W
BDT
PD48h
OPD
Fatty acid biosynthesis
Fabd
MECR
HSD17B8
ACACA
FASN
ACSL−1
ACSBG
ACSL−2
FabF
β−oxidation
HADHA
CPT1A
ACOX1−1
ACOX1−2
AtoB−1
ECI2
CPT2
GCDH
ACADVL
ECHS1
ACADSB
HADHB
ACADS
AtoB−2
ACADM
ACAA2
ECI1
Fatty acid elongation
HACD
ELOVL7−1
ELOVL7−2
HSD17B12−1
HSD17B12−2
TER
ELOVL7−3
PPT
Biosynthesis of unsaturated fatty acids
GPTE
npve
SCD
Arachidonic acid metabolism
PTGES3
CBR1
PTGES2
Lipid storage related protein
PLIN2−3
PLIN2−1
PLIN2−2
Lipid transport
FATP1
FATP4−2
FATP4−1
2
1
0
−1
−2
