# Supplementary material for: Comparative 4D Label-Free Quantitative Proteomic Analysis of Bombus terrestris Provides Insights into Proteins and Processes Associated with Diapause
Source: Int J Mol Sci. 2023 Dec 26;25(1):326. doi: 10.3390/ijms25010326 (PMC10778897; doi:10.3390/ijms25010326)
Supplement: Supplementary file 1 [file ijms-25-00326-s001.zip › Revised figures and supplementary materials/revised figures/Figure 7.pdf]

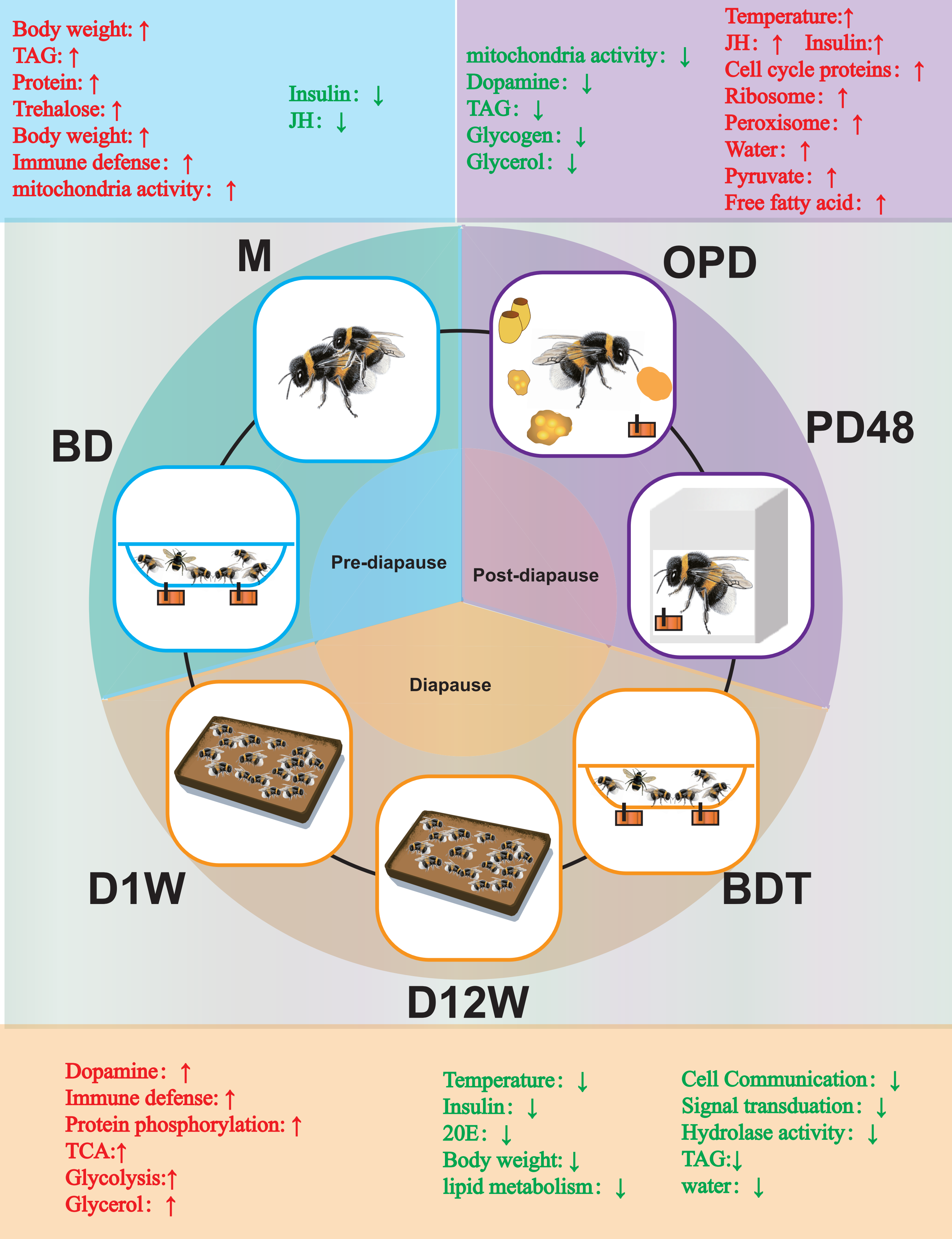
Body weight: ↑
TAG: ↑
Protein: ↑
Trehalose: ↑
Body weight: ↑
Immune defense: ↑
mitochondria activity: ↑
Insulin: ↓
JH: ↓
mitochondria activity: ↓
Dopamine: ↓
TAG: ↓
Glycogen: ↓
Glycerol: ↓
Temperature:↑
JH: ↑ Insulin:↑
Cell cycle proteins: ↑
Ribosome: ↑
Peroxisome: ↑
Water: ↑
Pyruvate: ↑
Free fatty acid: ↑
M
OPD
PD48
BD
Pre-diapause
Post-diapause
Diapause
D1W
BDT
D12W
Dopamine: ↑
Immune defense: ↑
Protein phosphorylation: ↑
TCA:↑
Glycolysis:↑
Glycerol: ↑
Temperature: ↓
Insulin: ↓
20E: ↓
Body weight: ↓
lipid metabolism: ↓
Cell Communication: ↓
Signal transduation: ↓
Hydrolase activity: ↓
TAG:↓
water: ↓
