# Supplementary figures and images for: Comparative 4D Label-Free Quantitative Proteomic Analysis of Bombus terrestris Provides Insights into Proteins and Processes Associated with Diapause
Source: Int J Mol Sci. 2023 Dec 26;25(1):326. doi: 10.3390/ijms25010326 (PMC10778897; doi:10.3390/ijms25010326)

A

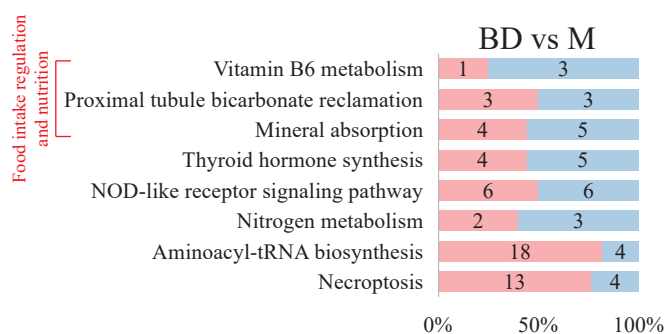

B

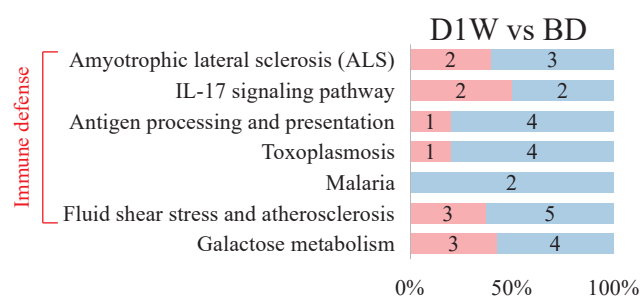

C

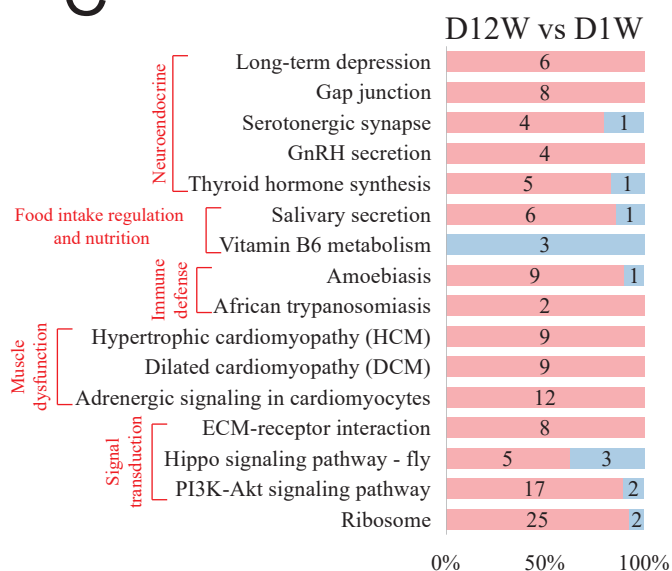

D

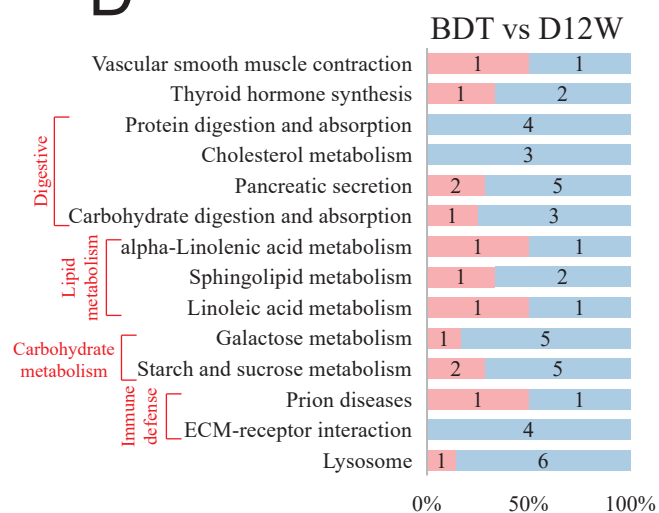

E

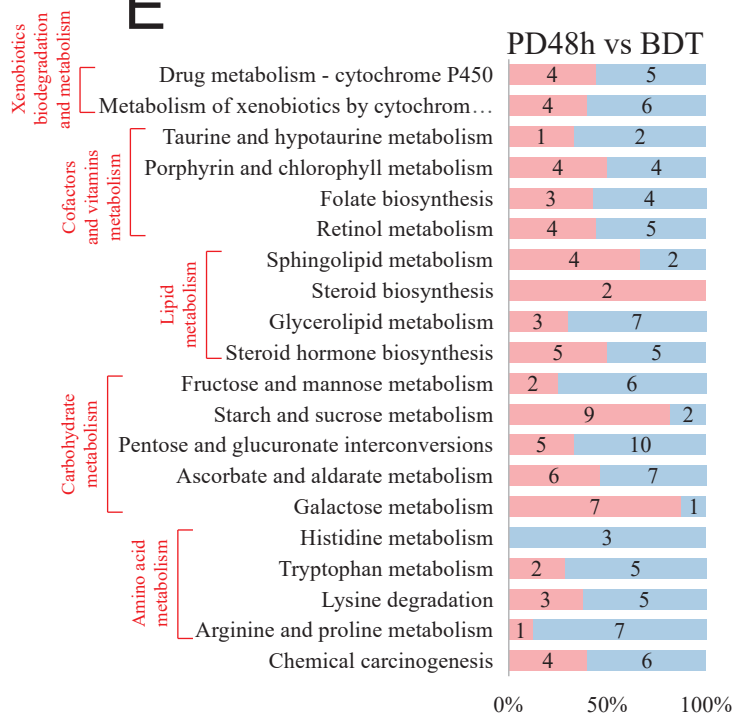

F

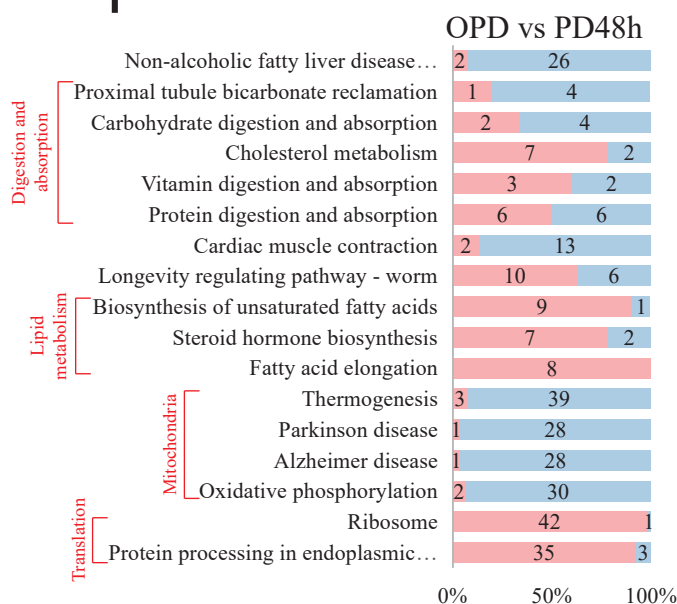

Up

Down

Supplement: Supplementary file 1 [file ijms-25-00326-s001.zip › Revised figures and supplementary materials/revised figures/Figure 2.pdf]

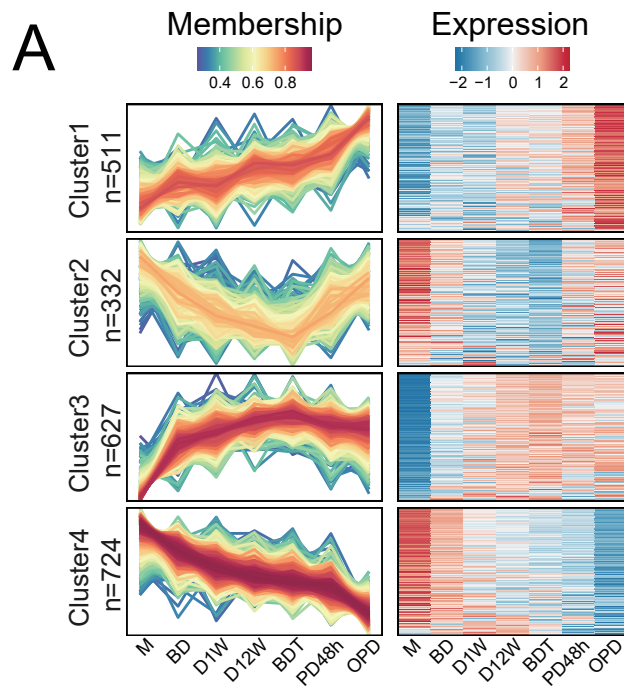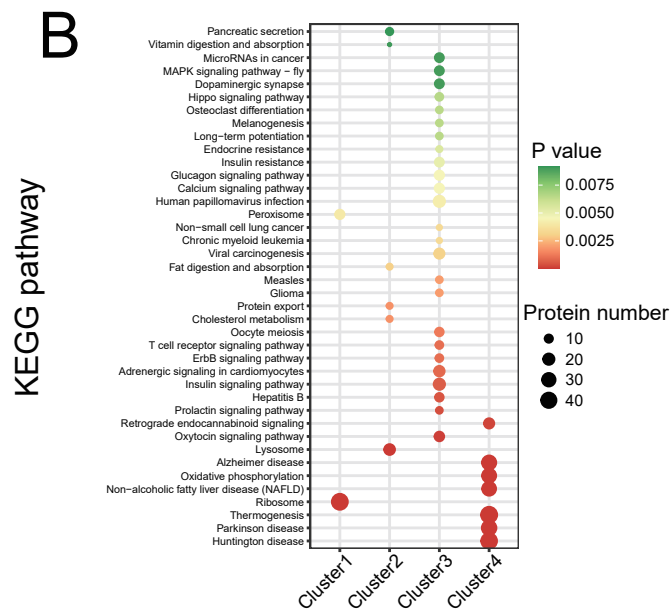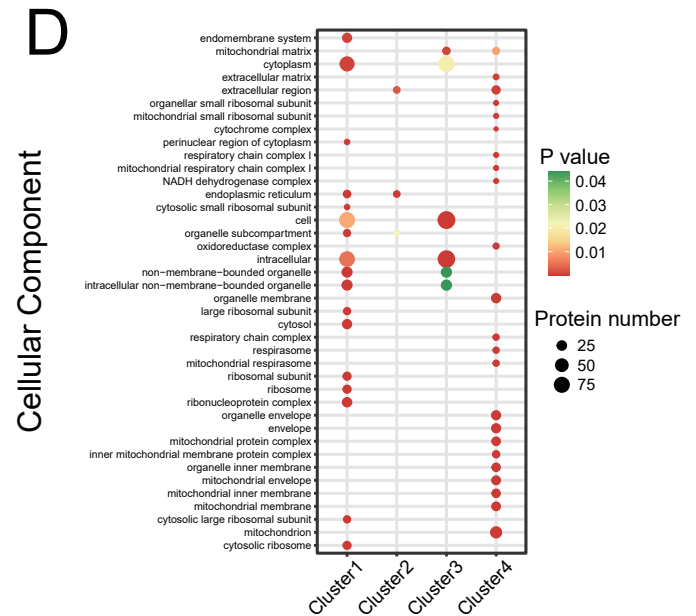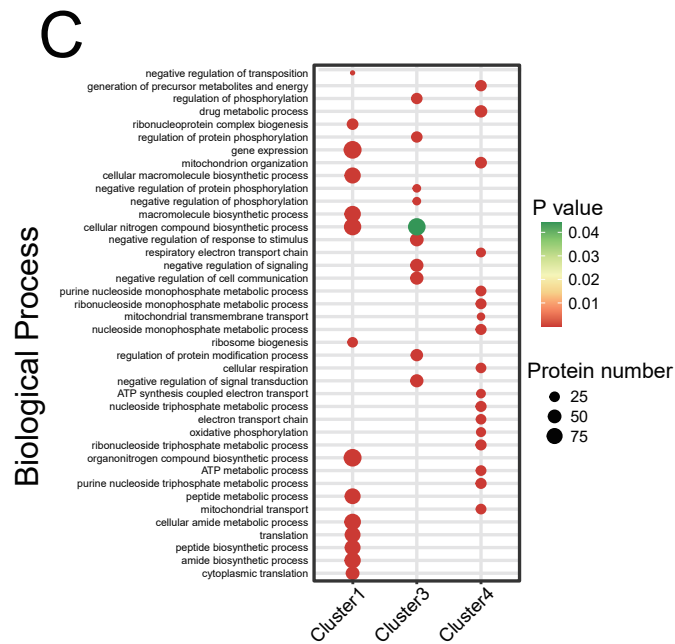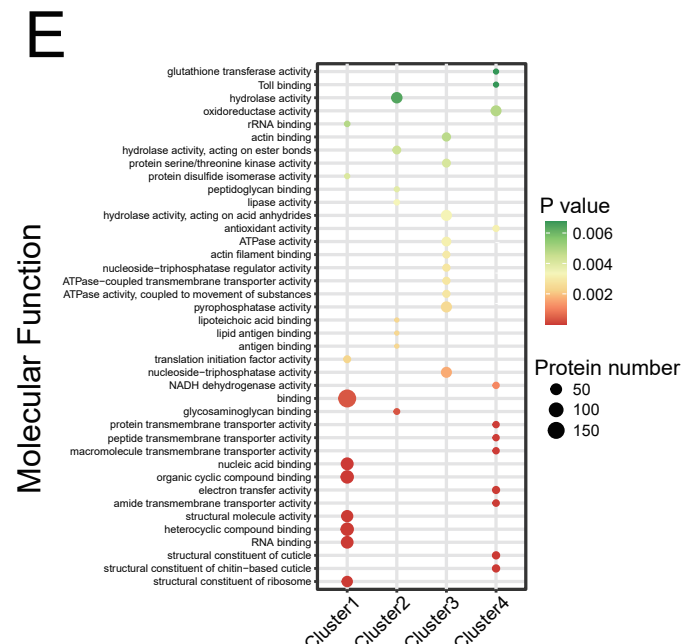

Supplement: Supplementary file 1 [file ijms-25-00326-s001.zip › Revised figures and supplementary materials/revised figures/Figure 3.pdf]

**A**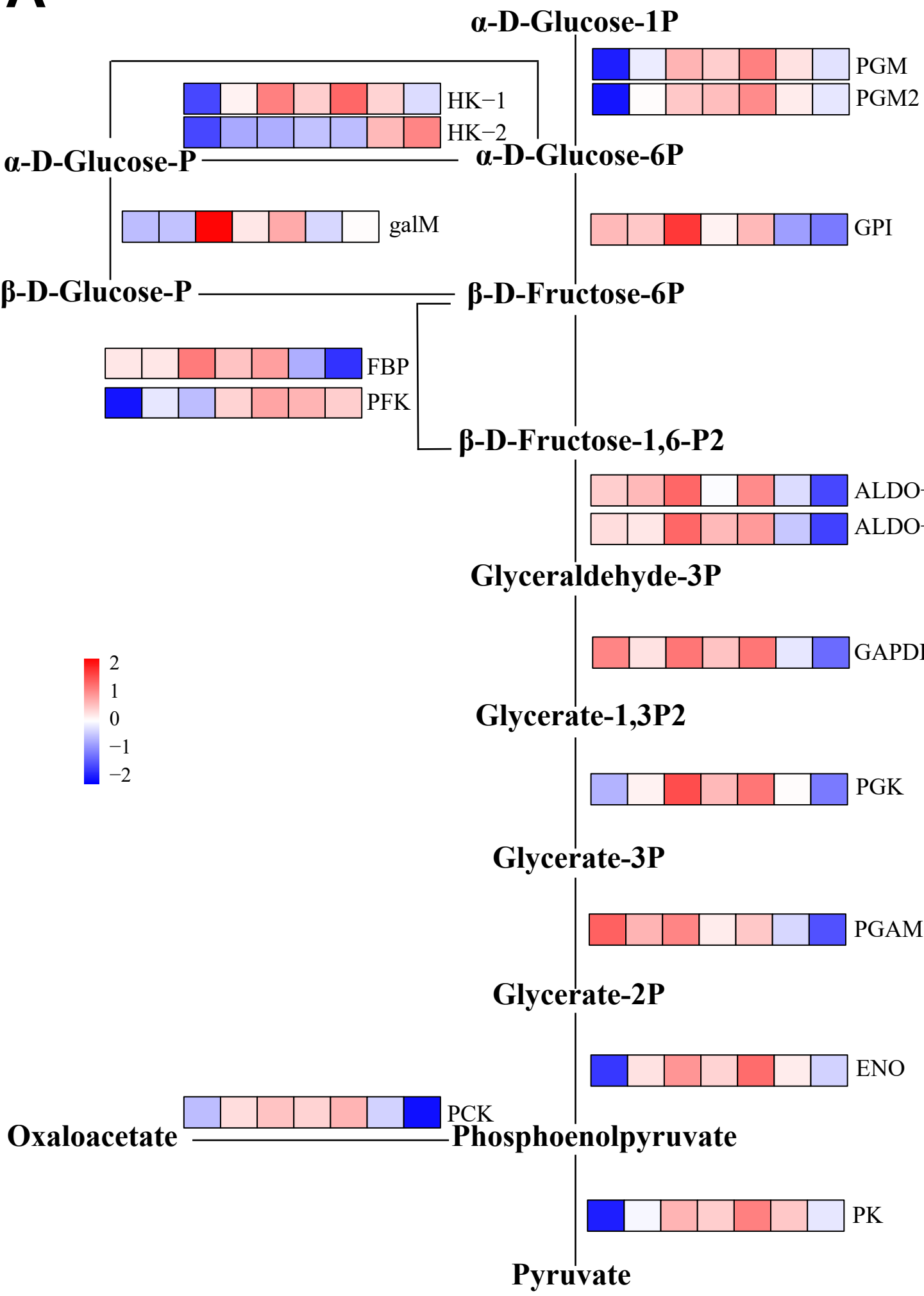**B**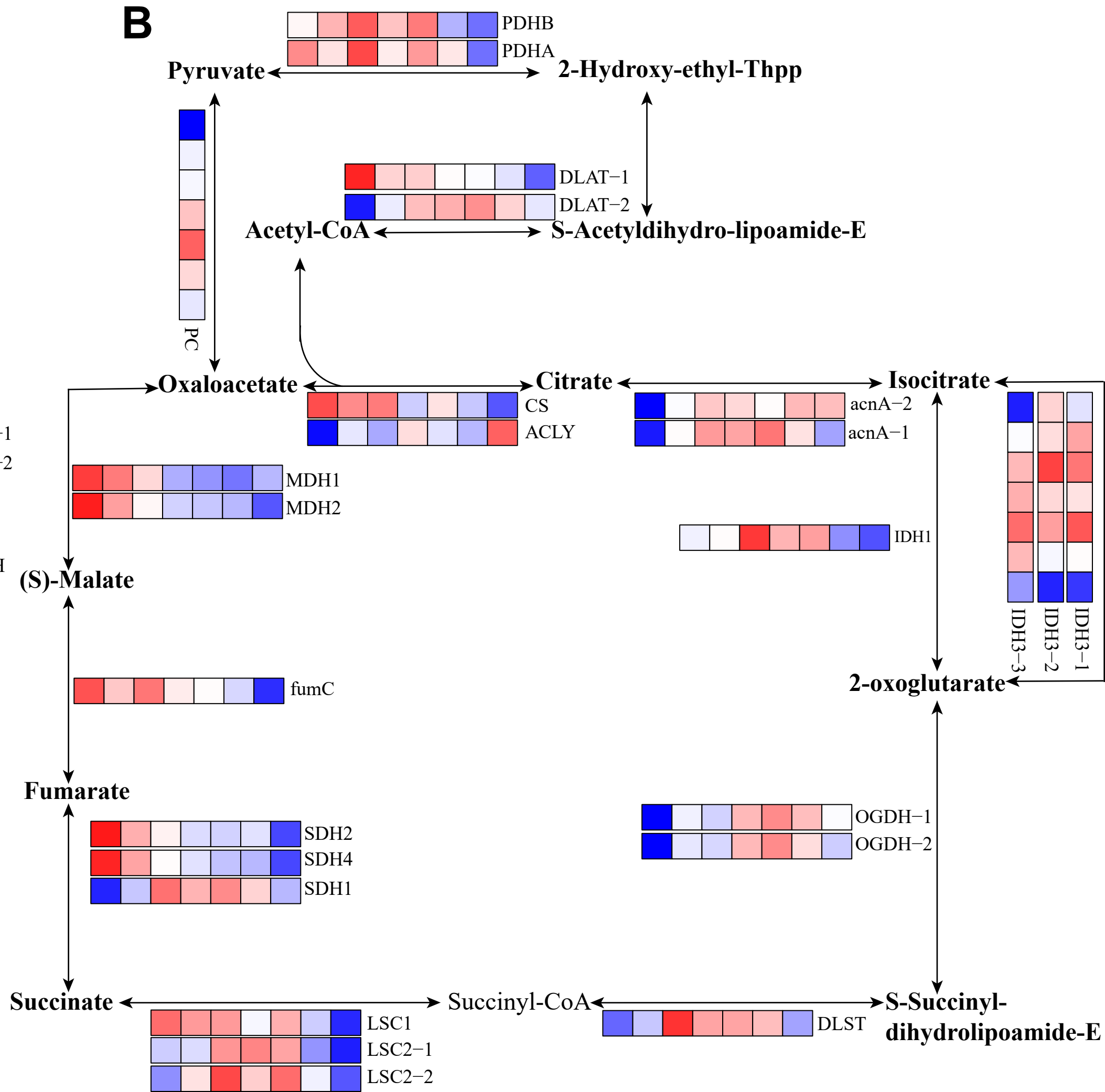

Supplement: Supplementary file 1 [file ijms-25-00326-s001.zip › Revised figures and supplementary materials/revised figures/Figure 5.pdf]

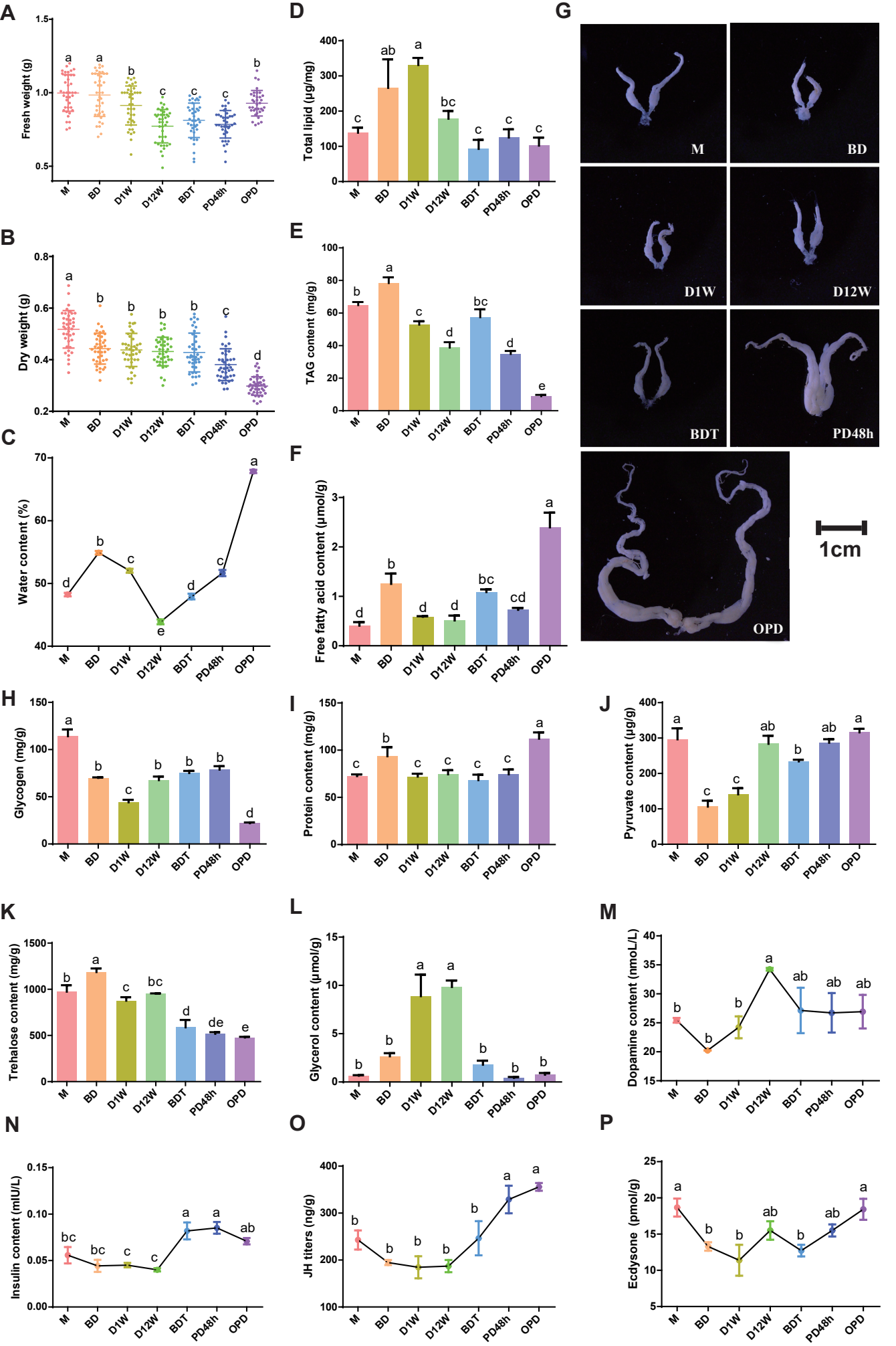

Supplement: Supplementary file 1 [file ijms-25-00326-s001.zip › Revised figures and supplementary materials/revised figures/Figure 6.pdf]

**A**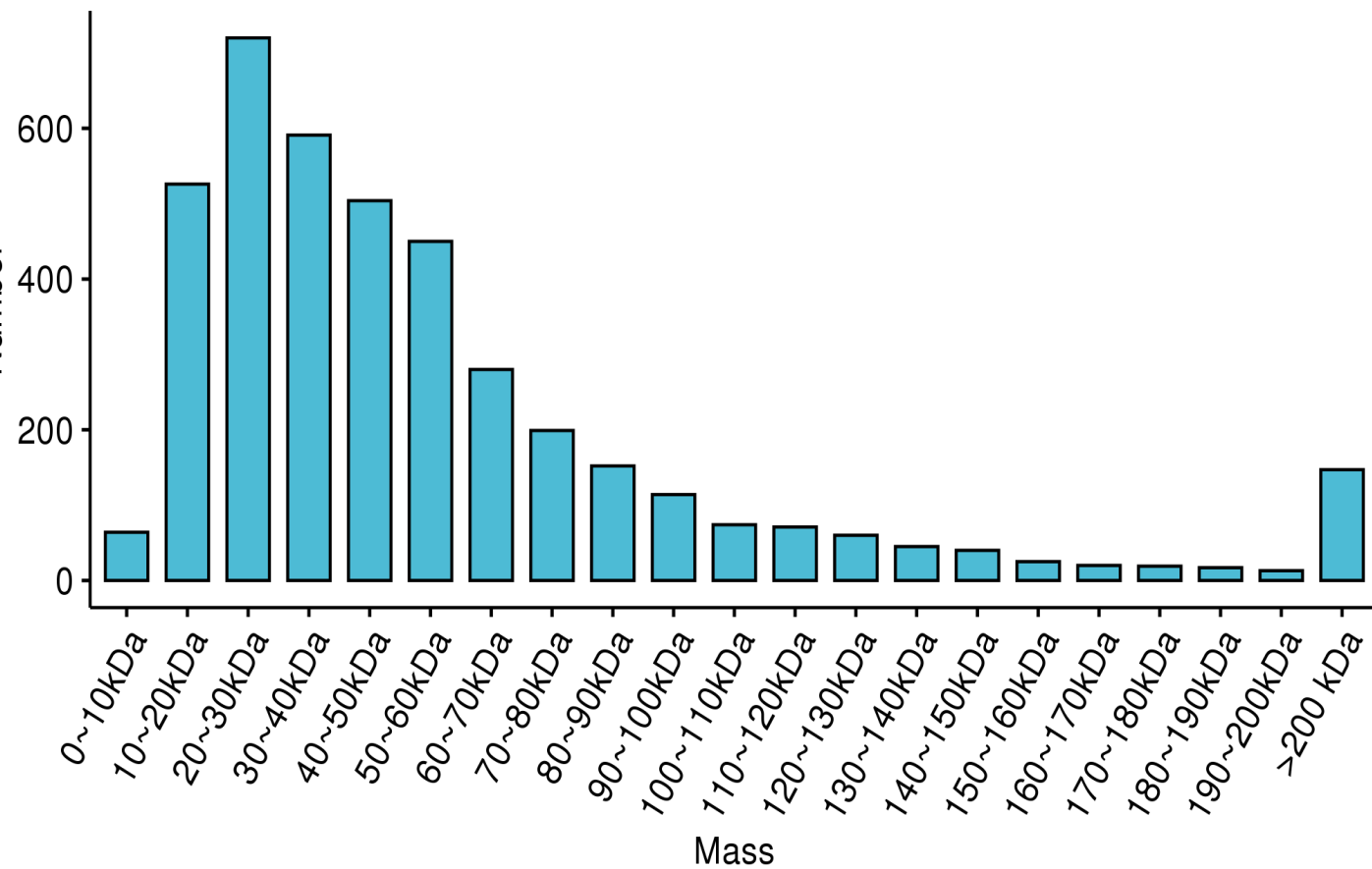**B**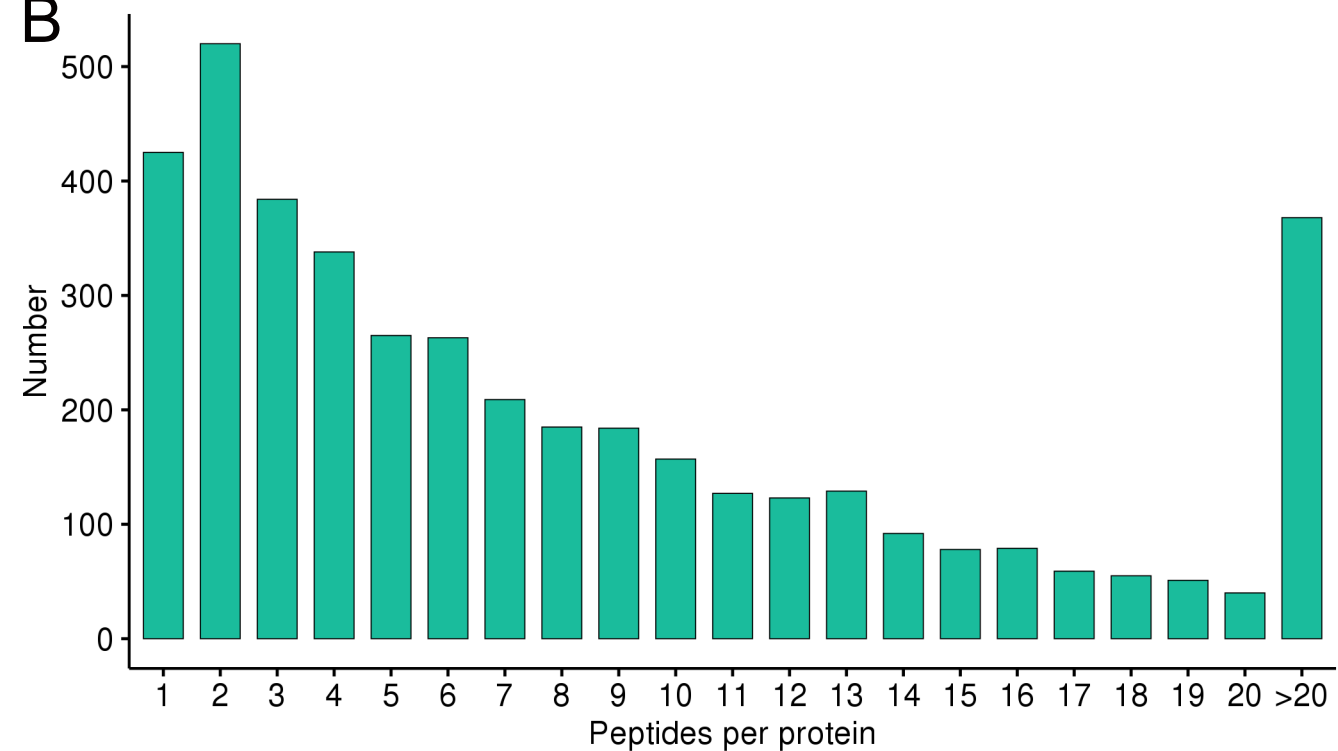

Supplement: Supplementary file 1 [file ijms-25-00326-s001.zip › Revised figures and supplementary materials/revised supplementary figures and tables/Supplementary figure 1.pdf]

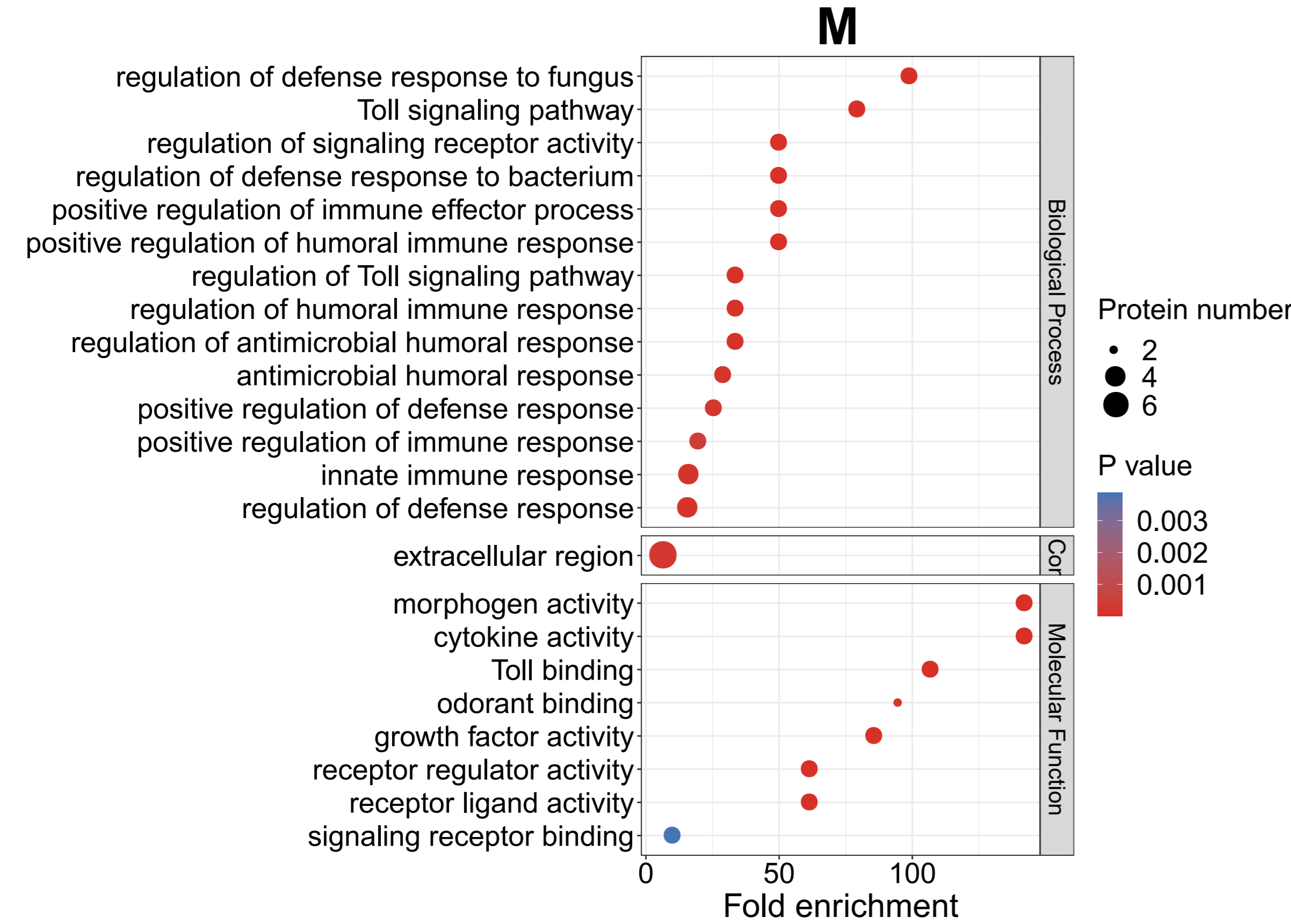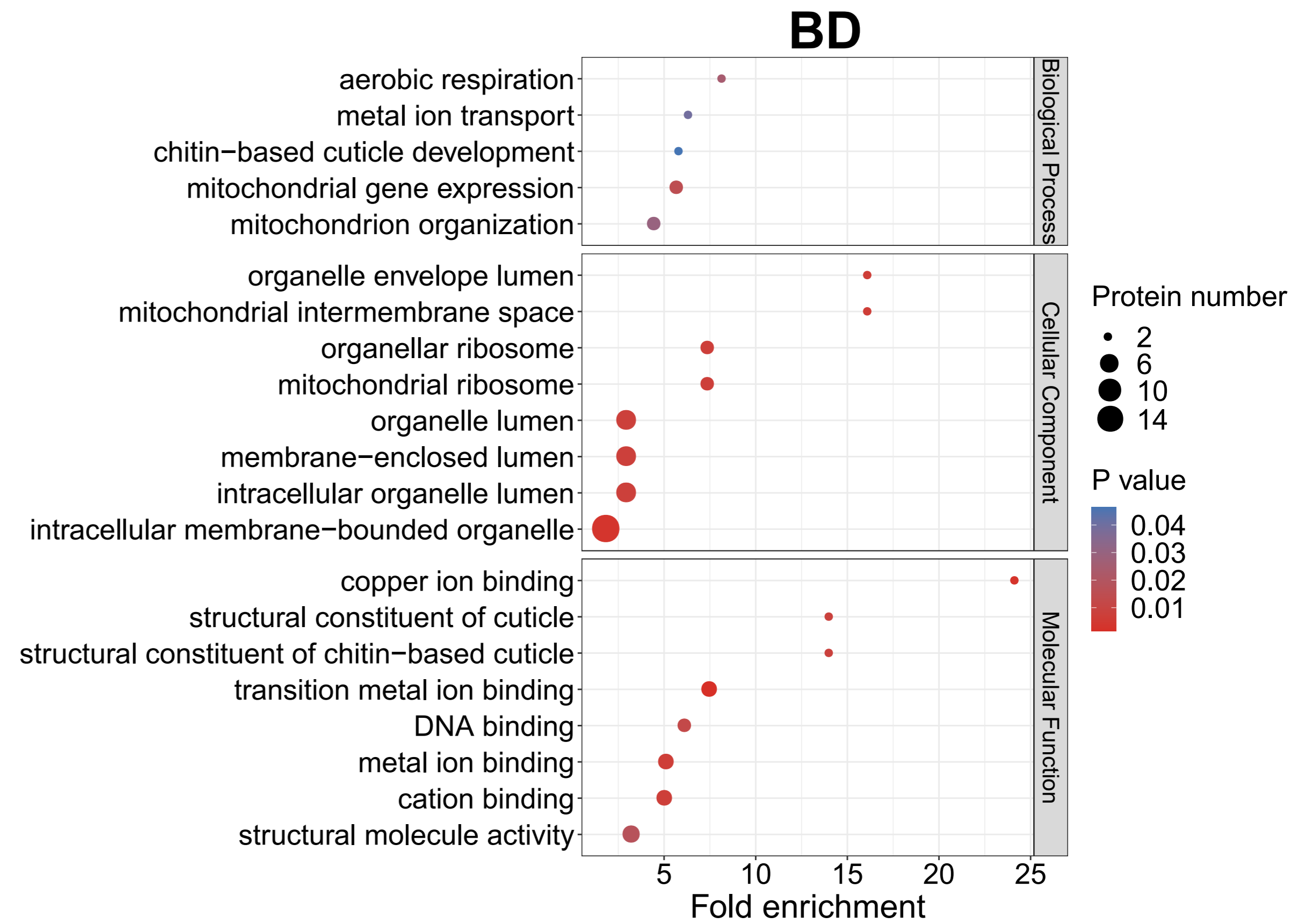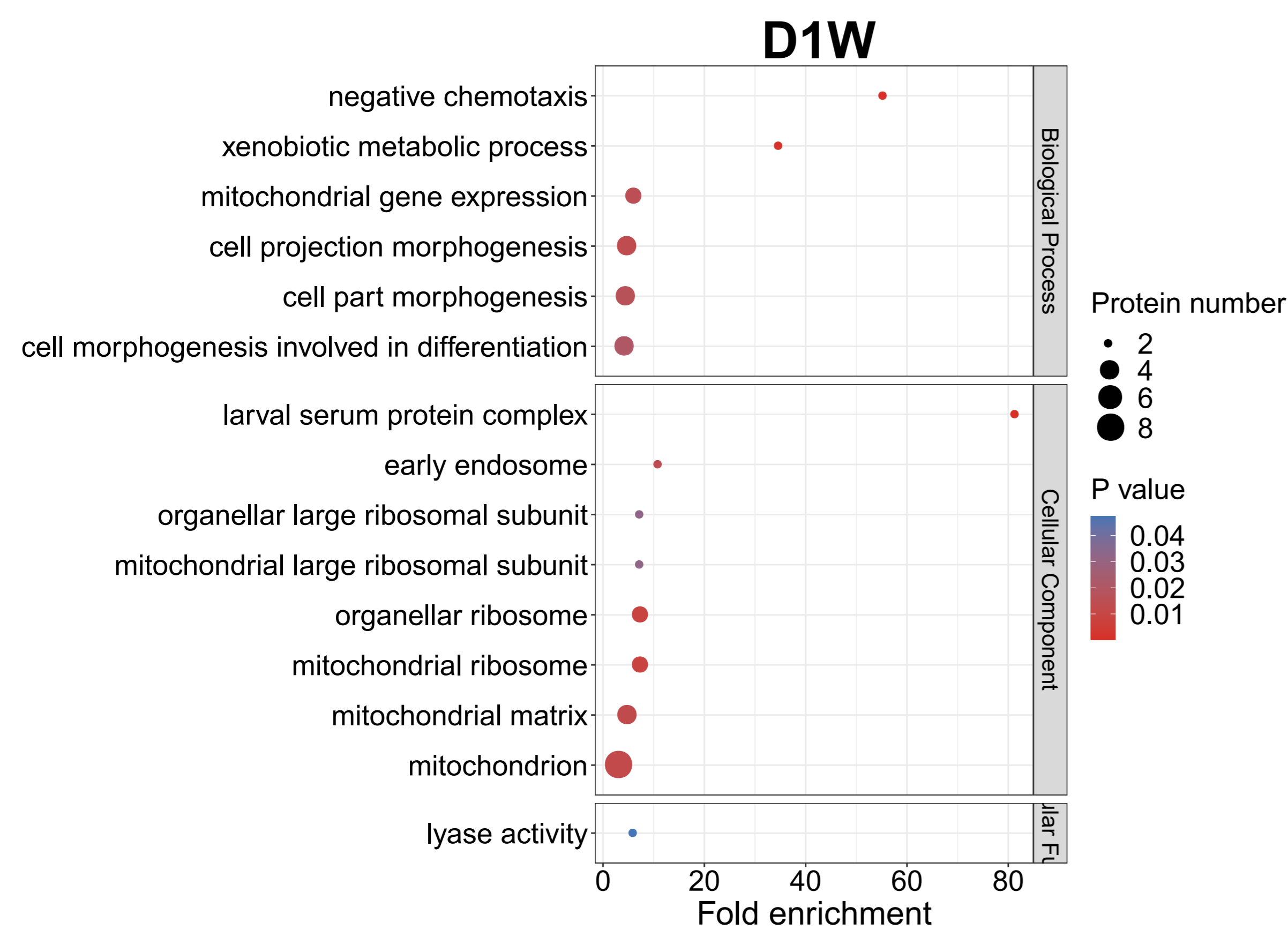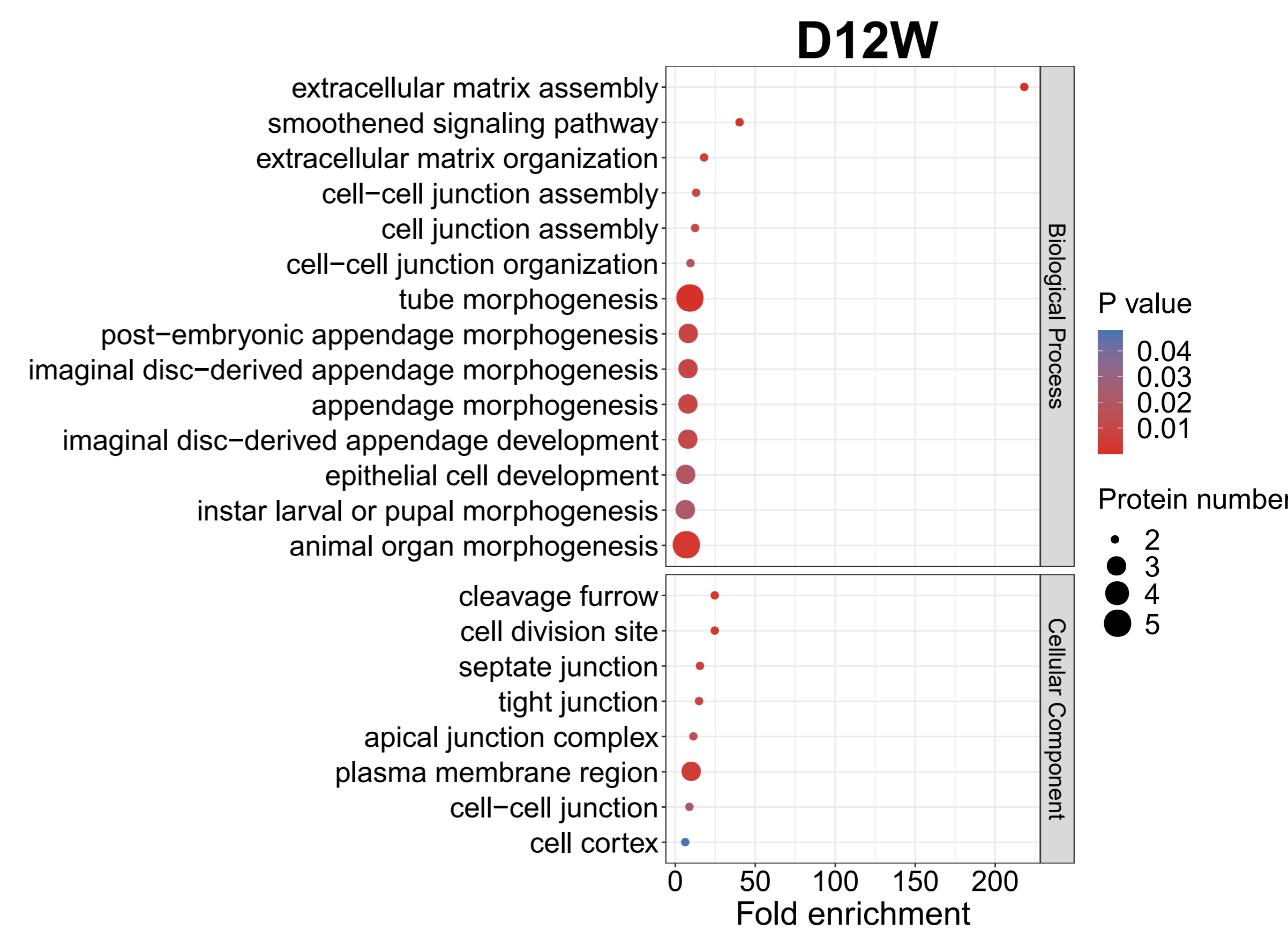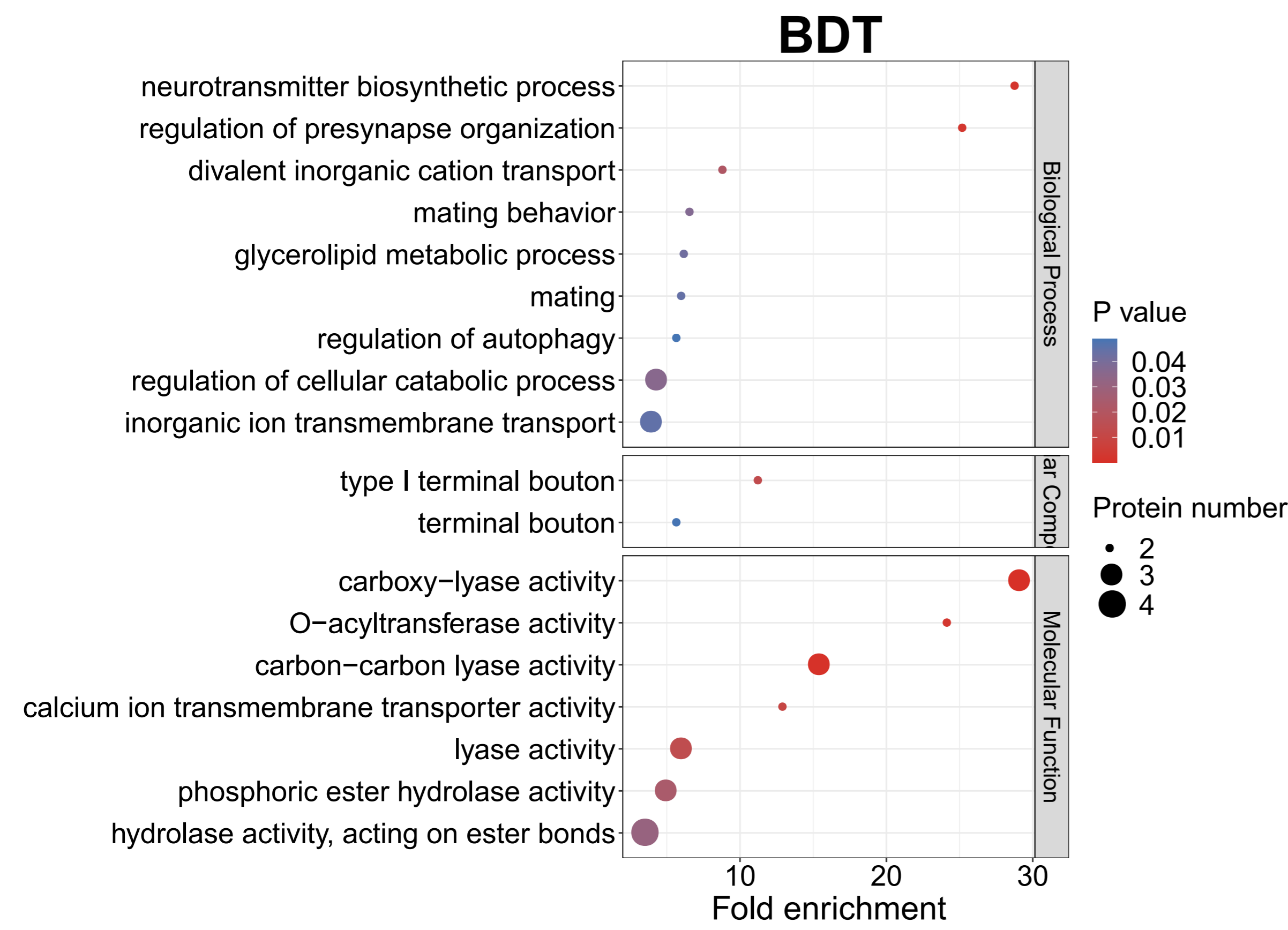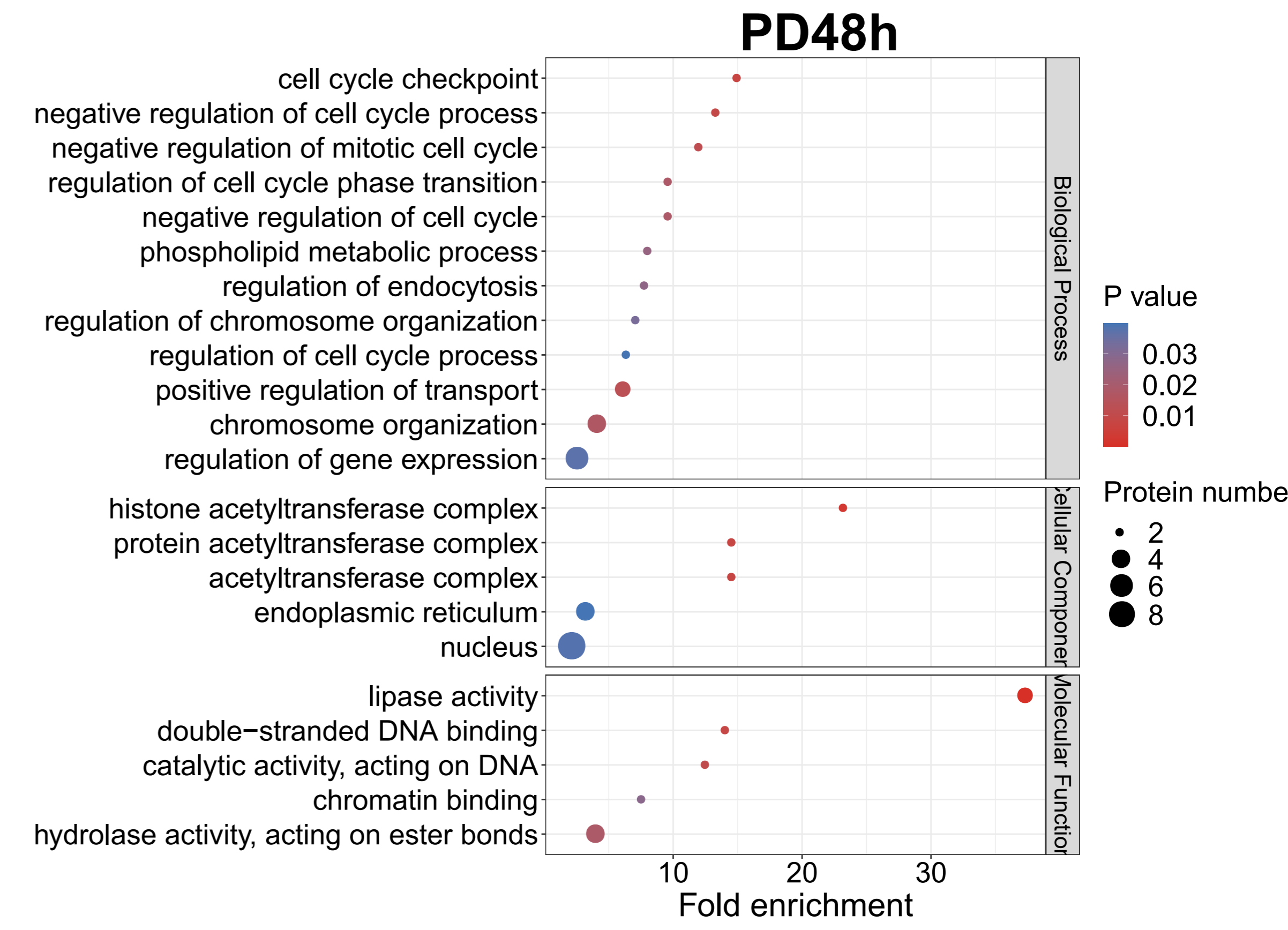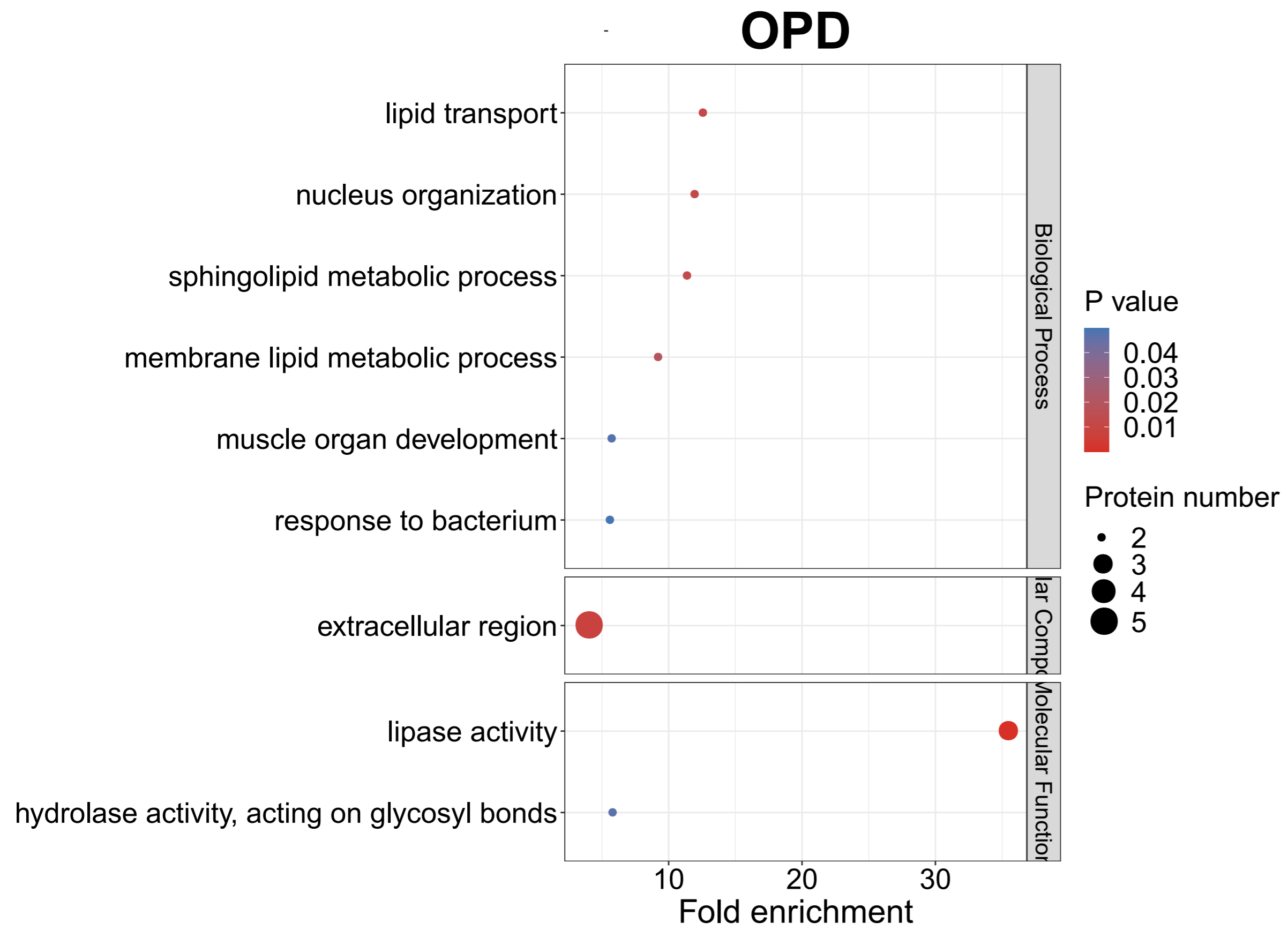

Supplement: Supplementary file 1 [file ijms-25-00326-s001.zip › Revised figures and supplementary materials/revised supplementary figures and tables/Supplementary figure 2.pdf]

## insulin signaling pathway

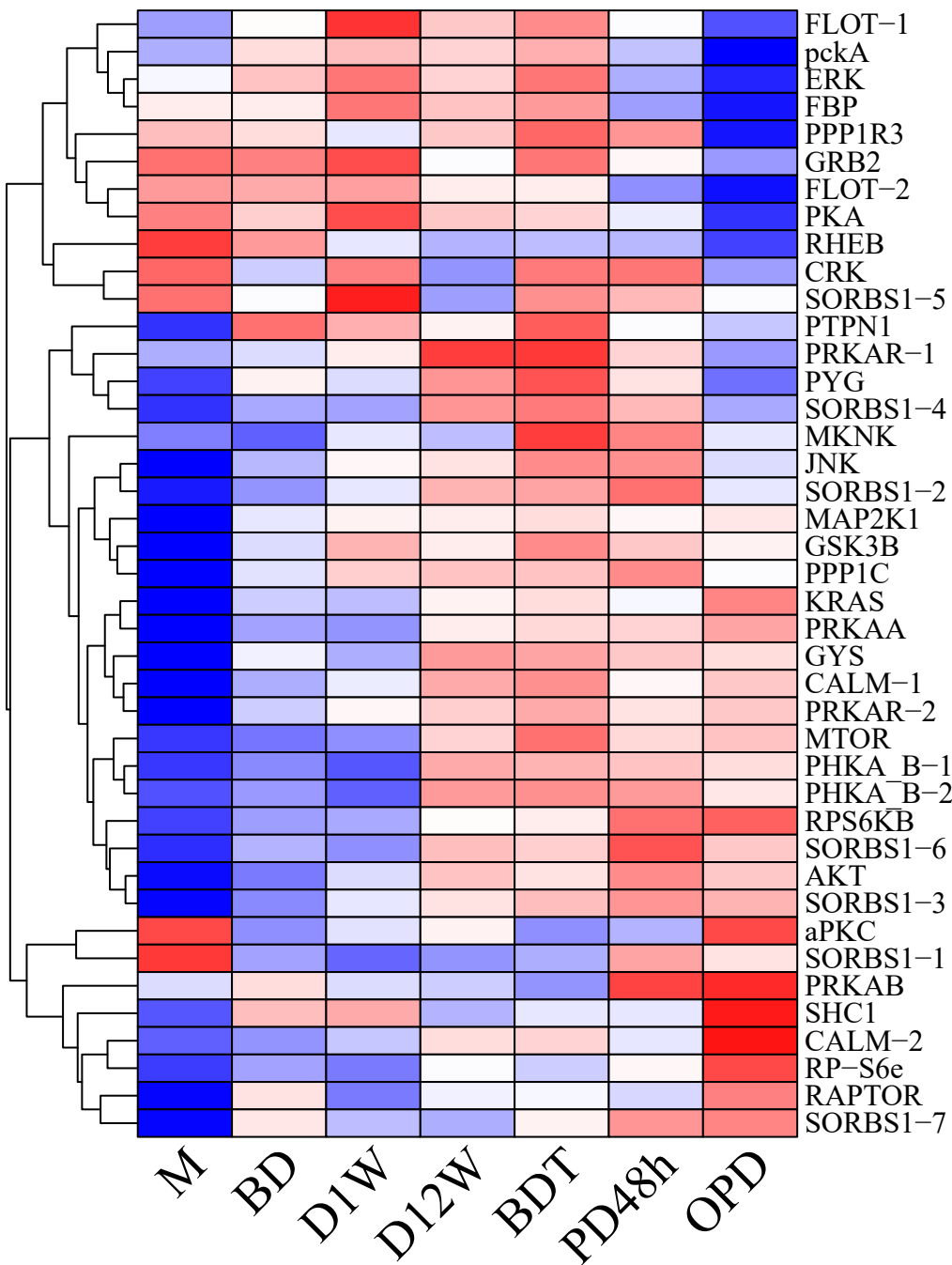

## dopaminergic synapse

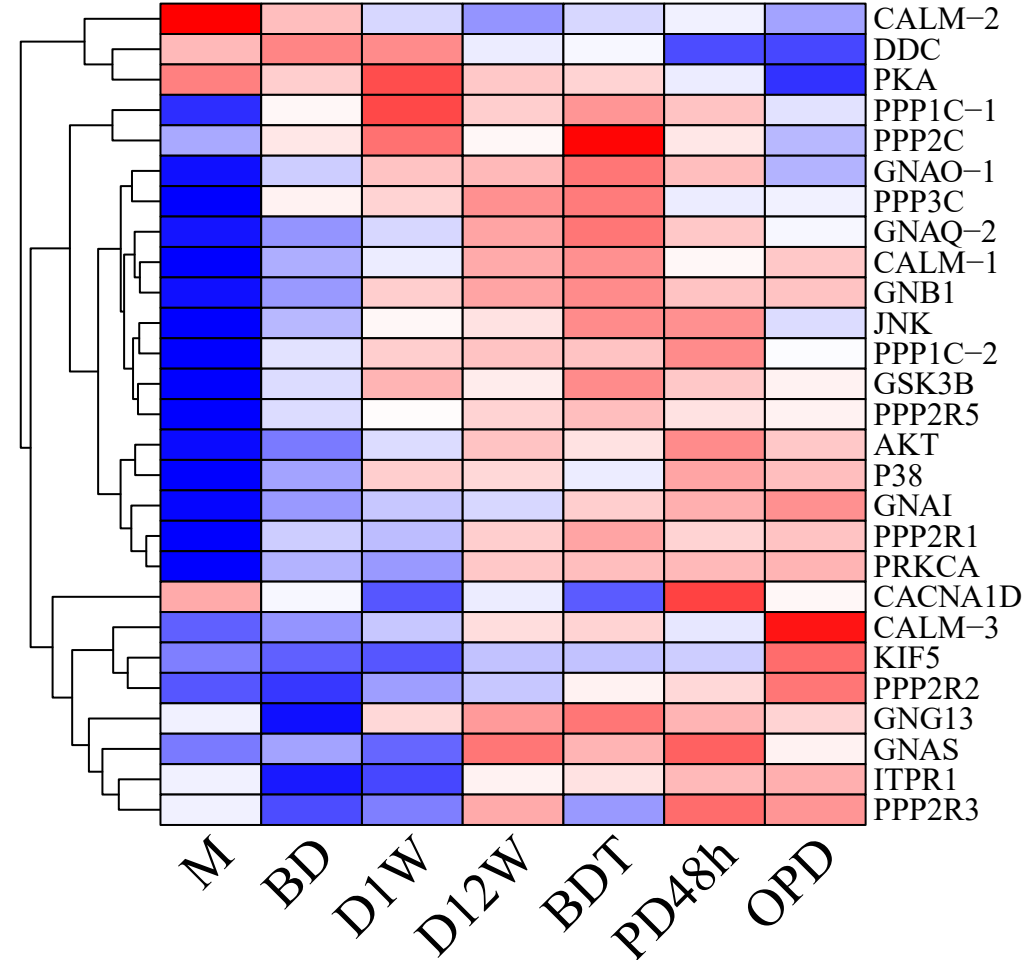

## JH

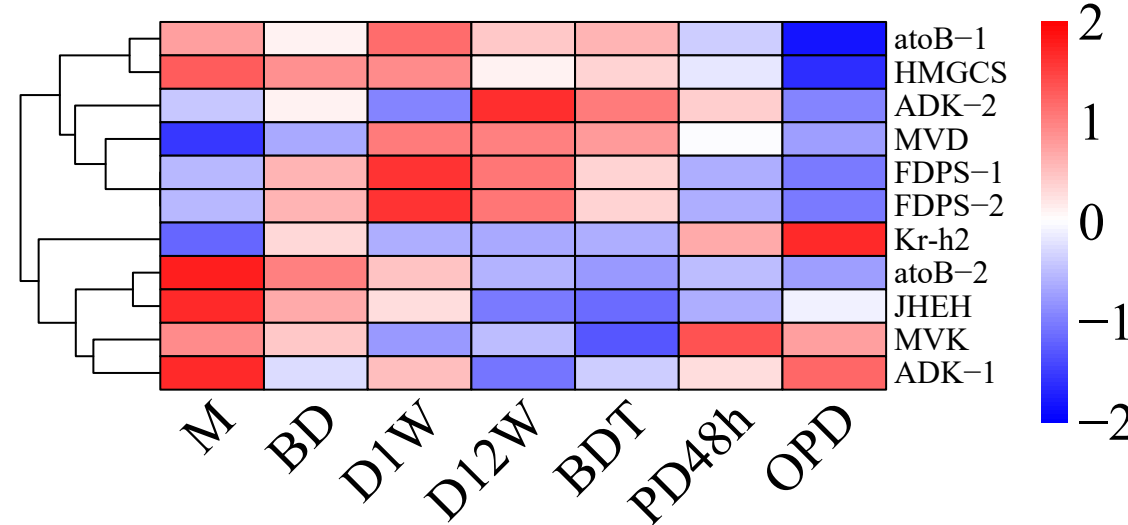

Supplement: Supplementary file 1 [file ijms-25-00326-s001.zip › Revised figures and supplementary materials/revised supplementary figures and tables/Supplementary figure 3.pdf]
